# Extracellular vesicles-mediated delivery of SpCas9 RNPs for therapeutic gene editing in Spinocerebellar Ataxia Type 3

**DOI:** 10.1016/j.biomaterials.2026.124119

**Published:** 2026-03-09

**Authors:** Kevin Leandro, David Rufino-Ramos, Sara M. Lopes, Francisca Santos Silva, Paulo Rodrigues-Santos, Ana Carolina Silva, Ana Rita Fernandes, Carina Henriques, Dina Pereira, Diana Lobo, Guilherme L. Gabriel, Rosário Faro, Rui Jorge Nobre, Pedro R.L. Perdigão, Benjamin P. Kleinstiver, Luís Pereira de Almeida

**Affiliations:** a CNC-UC - Center for Neuroscience and Cell Biology, University of Coimbra, Coimbra, Portugal; b CIBB - Center for Innovative Biomedicine and Biotechnology, University of Coimbra, Coimbra, Portugal; c Faculty of Pharmacy, University of Coimbra, Coimbra, Portugal; d Institute for Interdisciplinary Research, University of Coimbra, Portugal; e Institute of Immunology, Faculty of Medicine, University of Coimbra, Portugal; f GeneT - Gene Therapy Center of Excellence Portugal, University of Coimbra, Coimbra, Portugal; g Center for Genomic Medicine, Massachusetts General Hospital, Boston, MA, USA; h Department of Pathology, Massachusetts General Hospital, Boston, MA, USA; i Department of Pathology, Harvard Medical School, Boston, MA, USA; j ViraVector, University of Coimbra, Coimbra, Portugal

**Keywords:** Extracellular vesicles, CRISPR-Cas9, Gene editing, Machado-Joseph disease, Spinocerebellar ataxia type 3

## Abstract

Spinocerebellar Ataxia Type 3 (SCA3) is a neurodegenerative dominantly-inherited disorder caused by an overexpansion of a CAG tract within the *ATXN3* gene, conferring toxic properties to the ataxin-3 protein. Genome editing with CRISPR-Cas9 enzymes is a promising strategy to inactivate mutant *ATXN3* alleles, however, *in vivo* delivery remains challenging. Extracellular vesicles (EVs) are promising delivery vehicles for Cas9 and single guide RNA (sgRNA) ribonucleoproteins that minimize genomic exposure to highly active endonucleases.

In this study, we designed SpCas9 with a palmitoylation motif that enables SpCas9 and sgRNA enrichment into EVs. Introduction of a photocleavable linker - PhoCl - allowed the photo-inducible release of SpCas9 from the palmitoylation motif in EVs, increasing target engagement to *ATXN3 in vitro*. EVs loaded with SpCas9 ribonucleoproteins resulted in *ATXN3* knockout in SCA3 patient-derived iPSCs and two SCA3 animal models.

These findings highlight an innovative route for transient delivery of gene editing tools. This approach provides a promising therapeutic platform for the treatment of genetic diseases, including SCA3.

## 1. Introduction

Spinocerebellar Ataxia Type 3 (SCA3), also known as Machado-Joseph Disease (MJD), is a neurodegenerative dominantly inherited ataxia caused by an over-repetition of a CAG tract within the ataxin-3 gene (*ATXN3*) [1]. The expanded CAG trinucleotide repeats lead to an extended polyglutamine tract in the ATXN3 protein with toxic gain-of-function [2]. The mutant ATXN3 protein results in misfolding and aggregation in neural cells, ultimately leading to neuronal death in different brain regions, including the cerebellum, brainstem and striatum [2,3]. Targeting the upstream driver of SCA3 pathology, the *ATXN3* gene, is a promising therapeutic strategy to prevent the downstream toxic events resulting from the expression of mutant ATXN3 protein.

Clustered regularly interspaced short palindromic repeat (CRISPR)-associated protein (Cas) enzymes enable precise and efficient genome editing in human cells, allowing therapeutic editing for genetic diseases [4,5]. *Streptococcus pyogenes* Cas9 (SpCas9) nuclease contains two catalytic domains (HNH and RuvC) and is guided by a single guide RNA (sgRNA) to the target DNA sequence [4,6]. When engaged with a target DNA substrate, SpCas9:sgRNA ribonucleoprotein (RNP) complexes promote DNA double-strand breaks (DSBs) that can induce a frameshift in a gene coding sequence to cause premature stop codons [7,8]. Accordingly, this mechanism can be used to prevent the expression of disease-causing genes with a toxic gain-of-function [9]. Although genome editing with SpCas9 permits precise gene targeting, *in vivo* delivery is still a major bottleneck towards therapeutic applications due to size constraints, engagement to target cells and unwanted long-term exposure to editing enzymes [10].

Adeno-associated vectors (AAV) have been the leading vehicle for *in vivo* delivery of gene therapies, with several approved applications for gene replacement therapies [11,12]. However, AAVs present several limitations for the delivery of gene editing agents, including immunogenicity [13,14], limited DNA packaging capacity (*<*4.7 kb) [15], the prolonged expression of endonucleases which increases the likelihood of off-target mutagenesis [16], and can also result in the insertion of viral genomes in DSBs induced by Cas enzymes [17]. It is thus desirable to deliver Cas enzymes in a transient modality that allows gene editing and rapid turnover of active editing agents [18–20]. Delivery of Cas enzyme mRNA or RNPs offers an advantage by allowing potent on-target editing followed by rapid turnover of this enzyme in target cells [16,21,22].

Cell-derived vesicles, namely extracellular vesicles (EVs) and virus-like particles (VLPs), are natural carriers of nucleic acids and proteins, increasingly recognized as promising delivery vehicles for genome editing agents [21]. The nanometer size and cell surface-like membrane of EVs make them ideal for evading immune recognition and bypassing biological barriers. Through the expression of viral scaffolds, VLPs improve endosomal escape and cytosolic release of their cargo, as a tradeoff for increased immunogenicity compared to EVs. Cell-derived vesicles allow extensive engineering of their composition through the manipulation of their producer cells. Therapeutic cargos can be tethered to proteins naturally enriched in cell-derived vesicles to promote their endogenous loading into EVs and VLPs. Nevertheless, reversible dissociation between EV-enriched proteins and therapeutic cargos is desirable to promote packaging in producer cells and allow for nuclear delivery in target cells.

In this study, we engineered EVs and VLPs expressing VSV-G to encapsulate SpCas9 RNPs through the addition of a palmitoylation signal (Palm) to drive SpCas9 to the cell membrane. By engineering a photocleavable linker between Palm and SpCas9, we were able to induce the release of SpCas9 RNPs from the packaging Palm motif upon light exposure at 410 nm. SpCas9:sgRNA loaded EVs were shown to induce *ATXN3* knockout in cellular models, patient-derived iPSCs, and two SCA3 mouse models.

## 2. Results

### 2.1. Engineering SpCas9 RNP with a palmitoylation signal facilitates its enrichment in EVs

**Fig. 1.**
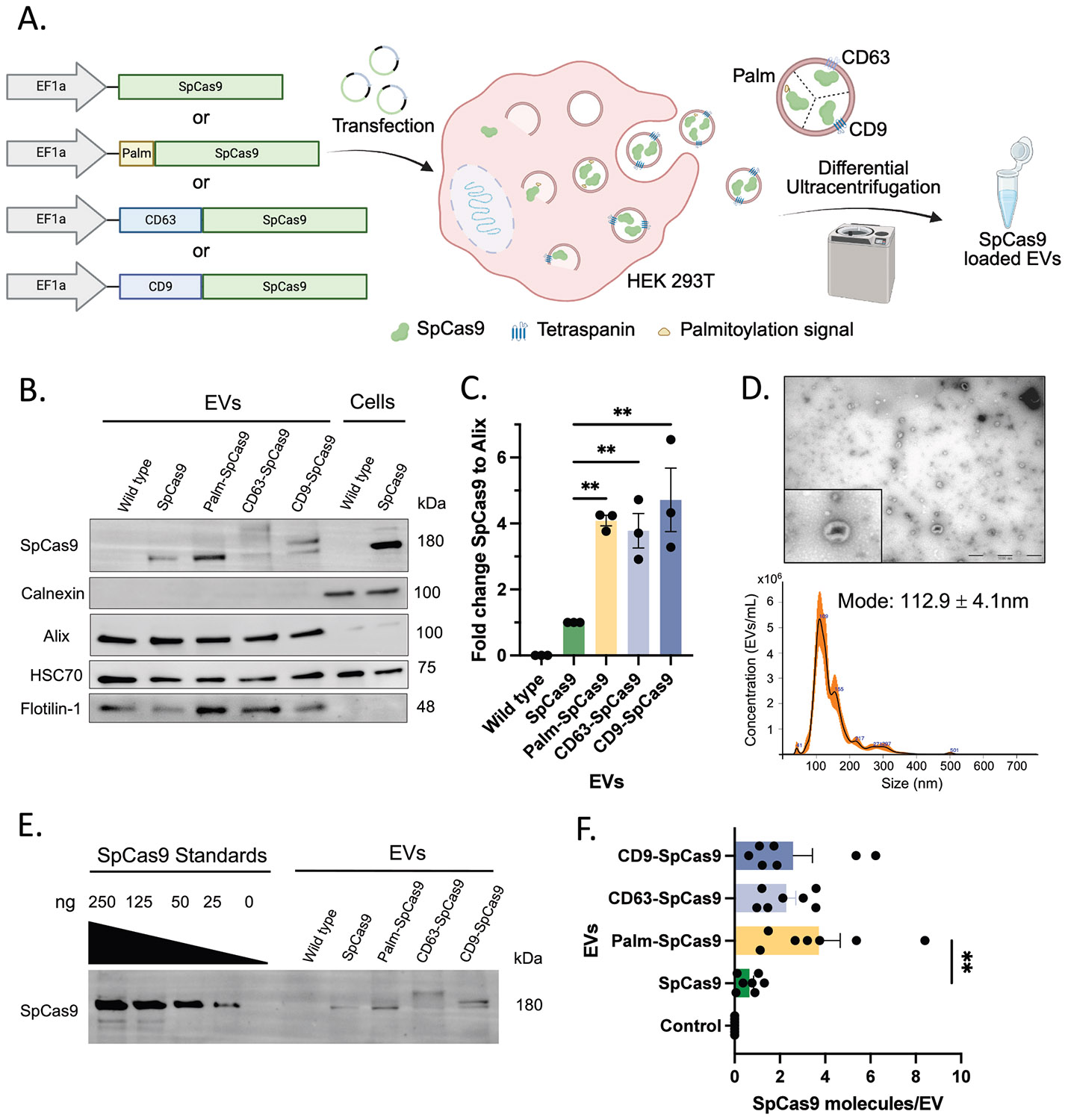
Engineering SpCas9 with a palmitoylation signal facilitates its enrichment in EVs. A. Schematic representation of SpCas9 enrichment in EVs through linking with Palm, CD63 or CD9 tetraspanins. Plasmid constructs are regulated by the EF1∝ promotor. Palm, CD63 and CD9 were fused to the N-terminal of SpCas9. Briefly, these constructs were transfected into HEK 293T producer cells, followed by EVs isolation via a differential ultracentrifugation protocol. B. Representative Western blot of EVs derived from HEK 293T cells overexpressing: SpCas9; Palm-SpCas9; CD63-SpCas9 or CD9-SpCas9 and wild-type EVs from HEK 293T cells; and cell lysates of wild-type and SpCas9 expressing cells. Membranes were immunolabelled with anti-SpCas9 antibody and with EV-positive markers Alix, HSC70, and Flotilin-1, as well as the EV-negative marker Calnexin. C. Levels of SpCas9 normalized to Alix by densitometric analysis, and the fold change relative to SpCas9 EVs was determined to assess SpCas9 enrichment (N = 3). Data are presented as mean ± SEM. Statistical analysis was performed with one-way ANOVA with Dunnett’s post hoc test. **p ≤ 0.01. D. The morphology of HEK 293T-derived EVs was evaluated by transmission electron microscopy (TEM) (scale bar represents 1000 nm), and the size and concentration by nanoparticle tracking analysis (NTA), showing a mode size of 112.9 ± 4.1 nm. E. Representative Western blot of SpCas9 purified protein standard curve, and EVs derived from cells expressing SpCas9 constructs. Membrane immunolabelled with anti-SpCas9 antibody. F. Densitometric analysis of a SpCas9 standard curve, combined with particle concentration measurements from NTA, was used to calculate the number of SpCas9 molecules per EV (N = 7). Data are presented as mean ± SEM. Statistical analysis was performed with one-way ANOVA with Dunnett’s post hoc test. **p ≤ 0.01.

We sought to develop a non-viral delivery platform based on EVs for the delivery of SpCas9 RNPs. To achieve this, we fused different EV packaging sequences to SpCas9 which, upon expression on producer cells, would facilitate the translocation of the SpCas9 protein from the cell cytoplasm into EVs. We tethered SpCas9 to three distinct EV packaging sequences: 1) tetraspanin CD9, 2) tetraspanin CD63 - two trans-membrane proteins highly enriched in EVs, particularly in exosomes [23] - and 3) a 10-amino acid palmitoylation signal (MLCCMRRTKQ), previously reported to mediate the association of proteins to cellular membranes and EVs [24]. In all three constructs, SpCas9 was placed at the C-terminus of the EV packaging protein, allowing for SpCas9 intraluminal localization within the EVs (Fig. 1A). To evaluate whether SpCas9 retained its nuclease activity while fused to these constructs, SpCas9, CD9-SpCas9, CD63-SpCas9, or Palm-SpCas9 plasmids were transfected in the presence or absence of a sgRNA targeting the *ATXN3* gene. The sgRNA KO.2 (from here on sgKO.2) was selected based on previous work [25]. Targeted amplicon sequencing was performed to determine the percentage of modified reads corresponding to sgKO.2 (Supplementary Fig. 1A). Western blot analysis showed that all fusion constructs markedly reduced ATXN3 protein levels (Supplementary Fig. 1B), indicating that SpCas9 nuclease activity was not compromised by fusion to Palm, CD9 or CD63.

EVs were produced and collected from HEK 293T cells transfected with Ef1*α*-driven constructs encoding SpCas9, CD9-SpCas9, CD63-SpCas9, or Palm-SpCas9 (Fig. 1A). EVs from non-transfected cells (wild type) were also collected to serve as a control. Western blot analysis of EV lysates revealed that all EV-types exhibited typical EV markers Flotilin-1, Alix, and HSC70, and were negative for the endoplasmic reticulum marker Calnexin (Fig. 1B). Moreover, EVs resulting from the overexpression of any of the four constructs were positive for SpCas9. To determine which packaging sequence promoted the highest enrichment, densitometric quantification of SpCas9 levels were normalized to Alix marker. Overexpression of Palm-, CD63-, or CD9-SpCas9 constructs in producer cells significantly enriched SpCas9 in EVs, resulting in 4.09-fold, 3.78-fold, and 4.72-fold increases, respectively, relative to control (Fig. 1C). In all conditions, the particles had a mean size ranging from 110 to 125 nm (Fig. 1D and Supplementary Fig. 2), which falls within the expected size for small EVs. HEK 293T derived EVs exhibited the typical cup-shaped morphology (Fig. 1D). To determine the number of SpCas9 molecules per EV in the different conditions, SpCas9 protein levels were measured through densitometric analysis and normalized against a standard curve of known amounts of purified SpCas9 protein (Fig. 1E). SpCas9 protein levels were further normalized to the total particle quantification determined by NTA (Fig. 1F). Palm-SpCas9, CD9-SpCas9 and CD63-SpCas9 mediated the highest enrichment of SpCas9 in EVs, with a mean of 3.72 ± 2.51, 2.59 ± 2.24 and 2.28 ± 1.12 SpCas9 molecules per particle, respectively (Fig. 1F). The Palm-SpCas9 construct was selected for downstream studies due to a significantly smaller size (~1.3 kDa) compared to the tetraspanin CD9 (~24 kDa), considering that palmitoylation is a reversible post-translational modification and its ubiquitous presence in all EV subtypes [26]. Overall, fusion of SpCas9 to a palmitoylation sequence results in higher loading of SpCas9 into EVs, thus establishing an effective packaging system to encapsulate SpCas9 protein in EVs.

### 2.2. Association of a cleavable linker to Palm-SpCas9 allows light-induced release of SpCas9 in EVs

**Fig. 2.**
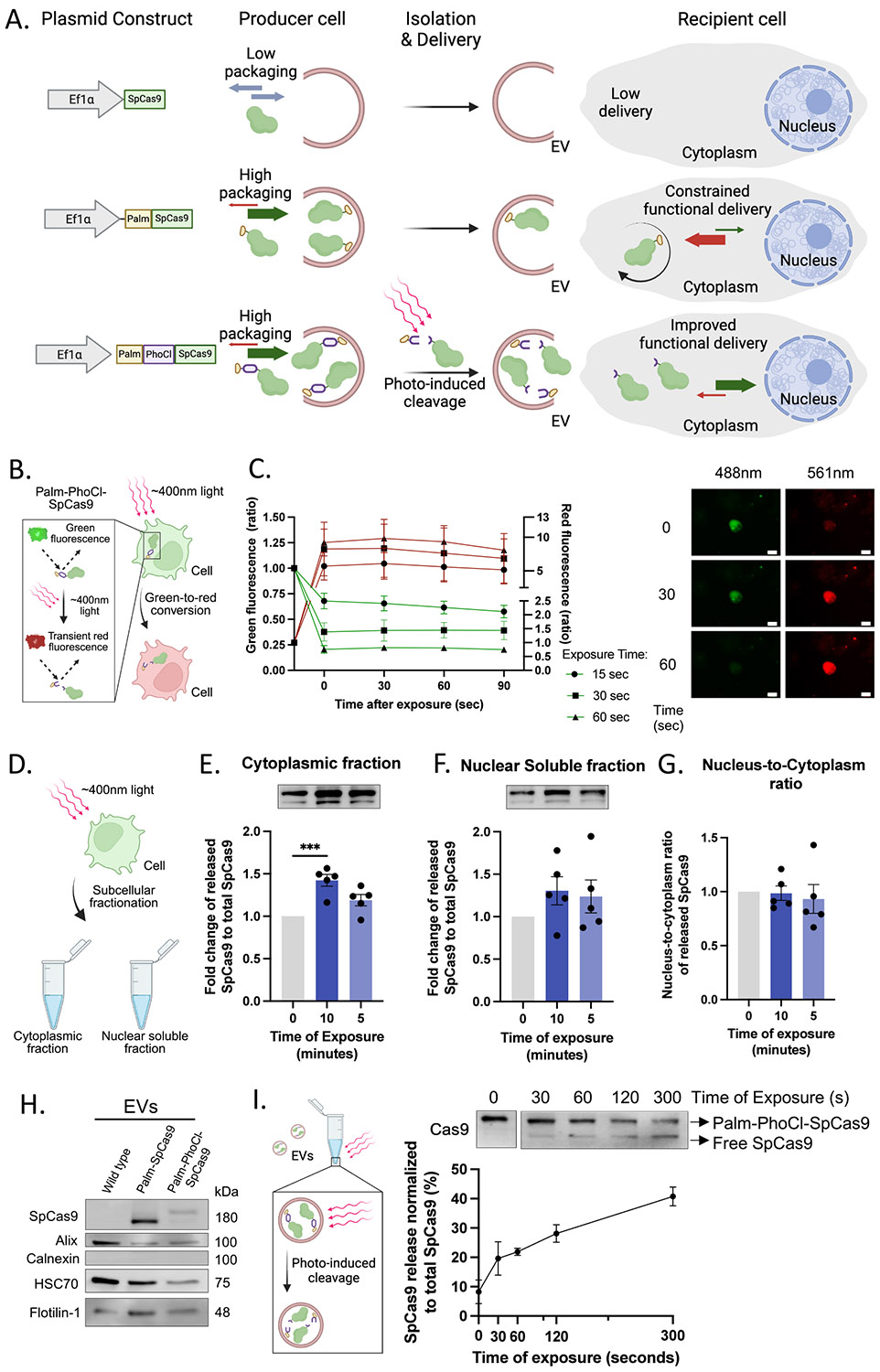
Association of a photocleavable linker to Palm-SpCas9 allows light-induced release of SpCas9 in EVs. A. Schematic representation of the introduction of the photocleavable linker PhoCl between the palmitoylation signal and SpCas9 sequences. In producer cells, Palm promotes the enrichment of SpCas9 in EVs. After isolation, EVs are exposed to 410 nm light to induce PhoCl cleavage and dissociation of SpCas9 from the EV packaging signal. Following delivery to recipient cells, free SpCas9 RNPs inside EVs can reach the nucleus of the target cell. B. Upon exposure to ~400 nm light, PhoCl is disrupted and undergoes a green-to-red fluorescence transition. Photo-inducible cleavage of Palm-PhoCl-SpCas9 can be monitored by green-to-red conversion in HEK 293T cells. C. Exposing Palm-PhoCl-SpCas9 expressing cells to ~400 nm light from 15 to 60 s increases green-to-red photoconversion as observed after quantification of fluorescence microscopy images (Zoe Fluorescent Cell Imager) (N = 5). Data are presented as mean ± SD. Fluorescence levels are normalized to fluorescence intensity before ~400 nm light exposure. Scale in microscopy images represents 25 *μ* m. D. Subcellular fractionation of cytoplasmic and nuclear soluble fractions from Palm-PhoCl-SpCas9 expressing cells following 5 or 10 min of exposure to ~400 nm light. E. Fold-change of released SpCas9 relative to total SpCas9 in the cytoplasmic fraction of cells exposed to ~400 nm light for 5 to 10 min (N = 5). Data are presented as mean ± SEM. Statistical analysis was performed with one-way ANOVA with Dunnett’s post hoc test. ***p ≤ 0.001. F. Fold-change of released SpCas9 relative to total SpCas9 in the nuclear soluble fraction of cells exposed to ~400 nm light for 5 to 10 min (N = 5). Data are presented as mean ± SEM. Statistical analysis was performed with one-way ANOVA with Dunnett’s post hoc test. No statistically significant differences were observed. G Nucleus-to-cytoplasmic ratio of released SpCas9 relative to total SpCas9 in cells exposed to ~400 nm light for 5 to 10 min (N = 5). Data are presented as mean ± SEM. Statistical analysis was performed with one-way ANOVA with Dunnett’s post hoc test. No statistically significant differences were observed. H. Representative Western blot of cells and EVs derived from cells expressing Palm-SpCas9 and Palm-PhoCl-SpCas9 showing SpCas9 protein enrichment in EVs. Membranes were immunolabelled with anti-SpCas9 antibody and with EV-positive markers Alix, HSC70, and Flotilin-1 and EV-negative marker Calnexin. I. Exposure of EVs to 410 nm light from 15 to 300 s increased the amount of released SpCas9 as observed by Western blot (N = 3). Data are presented as mean ± SEM.

As demonstrated above, the association of SpCas9 to a palmitoylation sequence results in SpCas9 enrichment in EVs in producer cells. However, upon delivery to recipient cells, palmitoylated SpCas9 might be recruited to the cell membrane and re-secreted in budding EVs [27], instead of being directed toward the nucleus (Fig. 2A). To prevent this, we aimed to incorporate a cleavable linker to dissociate SpCas9 from the Palm packaging domain after EV production and isolation (Fig. 2A). Here, we used a photocleavable protein (PhoCl) [28], previously described to spontaneously dissociate into two fragments upon ~400 nm light exposure [28], thus providing a light-mediated controllable release of SpCas9 from the Palm-motif in EVs *ex vivo*, prior to delivery in recipient cells (Fig. 2A).

First, we assessed the functionality of the Palm-PhoCl-SpCas9 construct in producer cells. Upon ~400 nm light exposure, PhoCl undergoes a green-to-red fluorescence transition due to photo-induced cleavage (Fig. 2B). Cells expressing Palm-PhoCl-SpCas9 were exposed from 15 to 60 s to ~400 nm light (Fig. 2C). As exposure to ~400 nm light increases, a decrease in green fluorescence is accompanied by an increase in red fluorescence (Fig. 2C). This was further confirmed by confocal microscopy, which demonstrated a green-to-red photoconversion upon cells targeted exposure to ~400 nm light (Supplementary Fig. 3).

We then aimed to evaluate if photo-induced cleavage of Palm-PhoCl-SpCas9 increases nuclear levels of free SpCas9 in producer cells. To do this, Palm-PhoCl-SpCas9 expressing cells were exposed to ~400 nm light for either 5 or 10 min and compared to unexposed cells (control). Cells were collected and subcellular compartments were isolated by subcellular fractionation assay (Fig. 2D). Levels of released SpCas9 increased by approximately 1.4-fold in cytoplasm and 1.3-fold in nucleus, respectively (Fig. 2E–G) compared to total SpCas9 levels upon ~400 nm light exposure. Statistical significance was observed between cells exposed to 10 min of ~400 nm light and unexposed cells (control) in the cytoplasmic fraction.

Then, we evaluated the packaging of Palm-PhoCl-SpCas9 in EVs. To accomplish this, Palm-SpCas9 and Palm-PhoCl-SpCas9 constructs were transfected in HEK 293T cells, and EVs were isolated as previously described. Characterization of EVs by Western blot confirms the packaging of Palm-PhoCl-SpCas9, along with presence for EV-markers Flotilin-1, Alix, and HSC70, and the absence of negative marker Calnexin (Fig. 2H). NTA analysis indicated that Palm-PhoCl-SpCas9 EVs had a mean particle size of 112.6 ± 3.5 nm (Supplementary Fig. 4). To demonstrate that Palm-PhoCl-SpCas9 is incorporated into EVs, and not the result of co-purification from differential ultracentrifugation, we performed iodixanol density gradient ultracentrifugation of Palm-PhoCl-SpCas9 EVs. Following centrifugation at 100,000g for 18h, ten fractions were collected and washed in PBS at 100,000g for 2h. Western blot analysis of the 10 fractions showed that SpCas9 co-localized with canonical EV markers HSC70 and flotillin-1 in intermediate density fractions (F6–F8), supporting EV-associated loading of SpCas9 (Supplementary Fig. 5). To investigate the release of SpCas9 from the Palm-PhoCl motif, Palm-PhoCl-SpCas9 EVs were exposed to 410 nm light for durations ranging from 30 to 300 s. Densitometric quantification of full-length and free SpCas9 protein showed a light-induced release of SpCas9 molecules, increasing from 8.23% at 0 s to mean values of 19.62%, 21.90%, 28.13%, and 40.73% at 30, 60, 120, and 300 s, respectively. (Fig. 2I).

Collectively, our results show that incorporating a photocleavable linker between SpCas9 and an EV-packaging motif allows controllable, light-induced release of SpCas9 from the packaging domain. Notably, photo-induced release of SpCas9 from isolated EVs can be achieved prior to delivery, eliminating the need for photoactivation of target cells or tissues.

### 2.3. Palm-PhoCl-SpCas9 expression results in the co-enrichment of sgRNAs in EVs

**Fig. 3.**
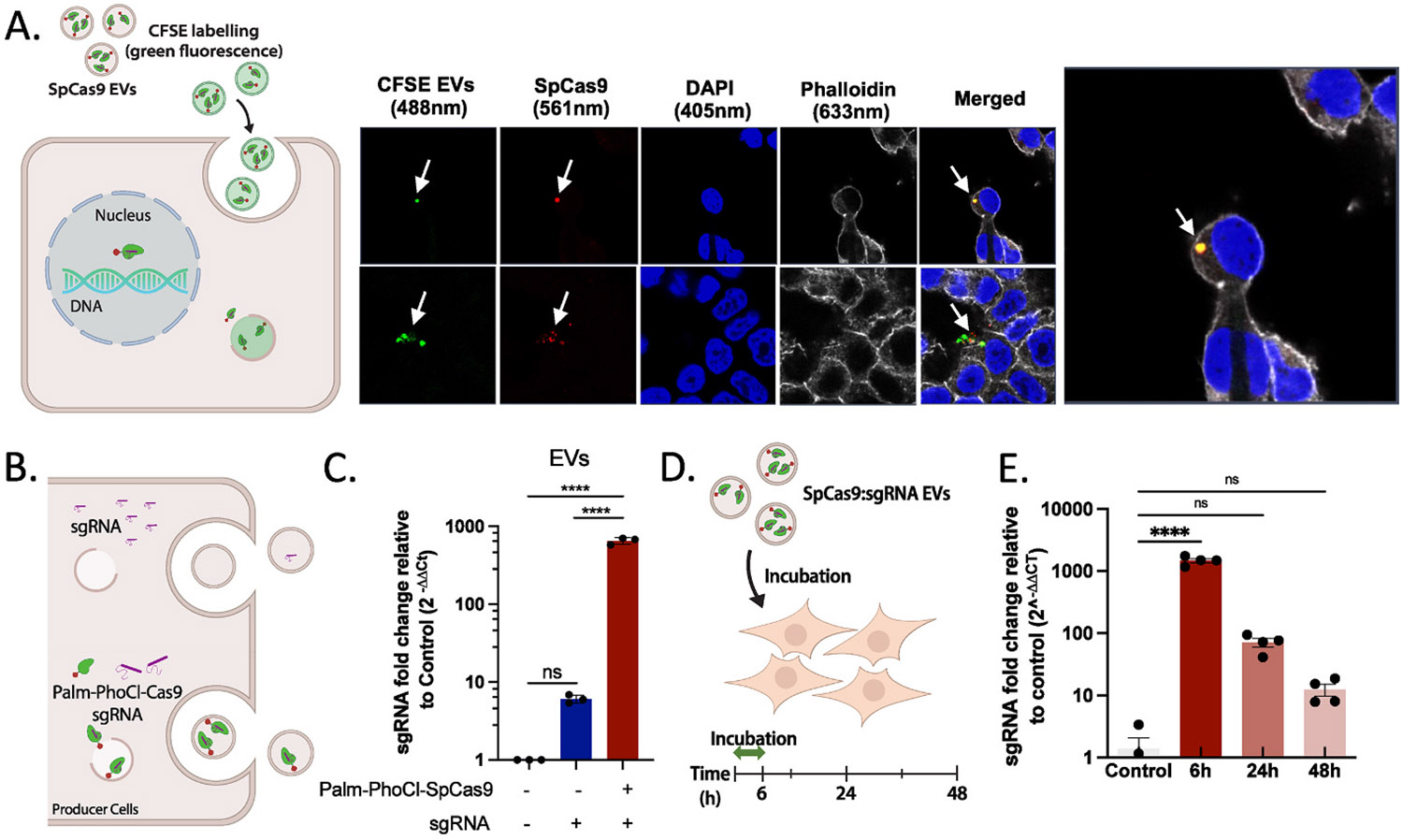
Palm-PhoCl-SpCas9 expression results in the co-enrichment of sgRNAs in EVs. A. CFSE-labelled Palm-PhoCl-SpCas9 EVs were incubated in HEK 293T cells for 6 h. Cells were collected and immunolabelled with antibody against flag (red), which is fused to SpCas9, and stained with DAPI (blue) and phalloidin (white) and analyzed by laser confocal microscopy to confirm EVs internalization. B. EVs were collected from HEK293T cells overexpressing sgKO.2 or both sgKO.2 and Palm-PhoCl-SpCas9 constructs. C. SgRNA enrichment in EVs was evaluated by RT-qPCR and normalized to GAPDH levels (N = 3). Data are expressed as mean ± SEM. A one-way ANOVA was performed to test multiple comparisons and post hoc Tukey’s test to examine differences between groups. ****p ≤ 0.0001. D. Palm-PhoCl-SpCas9:sgKO.2 EVs were incubated in HEK 293T cells for 6 h to evaluate the turnover of sgKO.2 at 6, 24 and 48 h. E. SgRNA levels in recipient cells were evaluated by RT-qPCR and normalized to GAPDH levels (N = 4). Data are expressed as mean ± SEM. Statistical analysis was performed with one-way ANOVA with Dunnett’s post hoc test. ****p ≤ 0.0001.

Following the enrichment and light-induced release of SpCas9 in EVs, our goal was then to evaluate the delivery of SpCas9 protein to recipient cells. EVs loaded with flag-tagged SpCas9 protein were labelled with CFSE, a green fluorescent intraluminal dye, and then incubated for 6 h in HEK 293T cells (Fig. 3A). After 24 h, cells were stained with anti-flag antibody for SpCas9 detection, DAPI, and phalloidin, and confocal microscopy was used to assess EVs internalization. Confocal microscopy confirmed the co-localization of CFSE-labelled EVs with SpCas9 anti-flag staining, suggesting EV internalization *in vitro* (Fig. 3A).

After confirming SpCas9 loading in EVs and its efficient delivery to recipient cells, we aimed at loading sgRNAs in EVs. To evaluate whether SpCas9 would facilitate the packaging of sgRNAs into EVs, HEK 293T cells were transfected either with sgKO.2 alone or combined with Palm-PhoCl-SpCas9 (Fig. 3B) and EVs were isolated as previously described. Following RNA extraction and cDNA synthesis, the amount of sgRNA enrichment was evaluated by RT-qPCR (Fig. 3C). Transfecting HEK 293T cells with the sgKO.2 encoding plasmid alone led to detectable amounts of sgRNA in EVs. In contrast, co-expressing sgKO.2 with Palm-PhoCl-SpCas9 resulted in a 90-fold enrichment of sgRNAs compared to EVs derived from cells expressing sgKO.2 plasmid alone (Fig. 3C). We hypothesized that sgRNAs are co-enriched into EVs due to the formation of a SpCas9:sgRNA RNP complex that protects and drives sgRNAs into EVs in producer cells. To evaluate the delivery and stability of sgRNA in recipient cells, EVs loaded with Palm-PhoCl-SpCas9:sgRNA RNP complex were incubated for 6 h in HEK 293T, and cells were collected after 6, 24 and 48 h (Fig. 3D). RT-qPCR analysis of recipient cells confirmed the presence of sgRNAs, showing a time-dependent decrease of sgRNA levels from 6 to 48 h after EVs incubation (Fig. 3E).

Overall, co-expression of Palm-PhoCl-SpCas9 and sgKO.2 in producer cells results in the enrichment of SpCas9:sgKO.2 RNPs in EVs, thus establishing an EV-packaging system that results in transient delivery of the genome editing tools *in vitro*.

### 2.4. Light-activated EVs loaded with Palm-PhoCl-SpCas9 RNPs reduce ATXN3 expression in vitro

After generating a packaging system to load EVs with SpCas9 protein and sgRNA, we aimed to evaluate *in vitro* efficiency in the context of monogenic disorders, particularly those where gene knockout would decrease the expression of the pathological protein. Here, we focused on SCA3, a neurodegenerative disease caused by a CAG overexpansion in exon 10 of the *ATXN3* gene [1,2].

We tested the efficacy of EV-mediated delivery of CRISPR-Cas9 using a previously validated sgRNA that targets exon 2 of *ATXN3* (sgKO.2) [25], inducing DSBs to cause frameshifts that generate a premature stop codon. To perform an initial assessment of off-target (OT) activity, four OT sites with ≤3 mismatches were nominated using Cas-OFFinder (Supplementary Table 2) and these genomic loci were PCR amplified and sequenced. High on-target editing efficiency was observed at the *ATXN3* locus in treated HEK 293T cells, with insertion or deletion mutation (indel) efficiencies of approximately 70% relative to an untreated control. Among the Cas-OFFinder nominated OT sites, OT site 1 resulted in ~2.4% higher levels of indels compared to background control levels (Supplementary Fig. 6). This OT site is in an intronic region and thus unlikely to interfere with protein-coding sequences in the human genome. Analysis of editing at OT sites 2-4 revealed similar levels of indels compared to control samples (Supplementary Fig. 6). Although no concerning OT events were observed in this initial dataset, more comprehensive OT nomination methods (such as GUIDE-seq, CHANGE-seq, etc.) should be employed for a thorough and unbiased OT assessment.

**Fig. 4.**
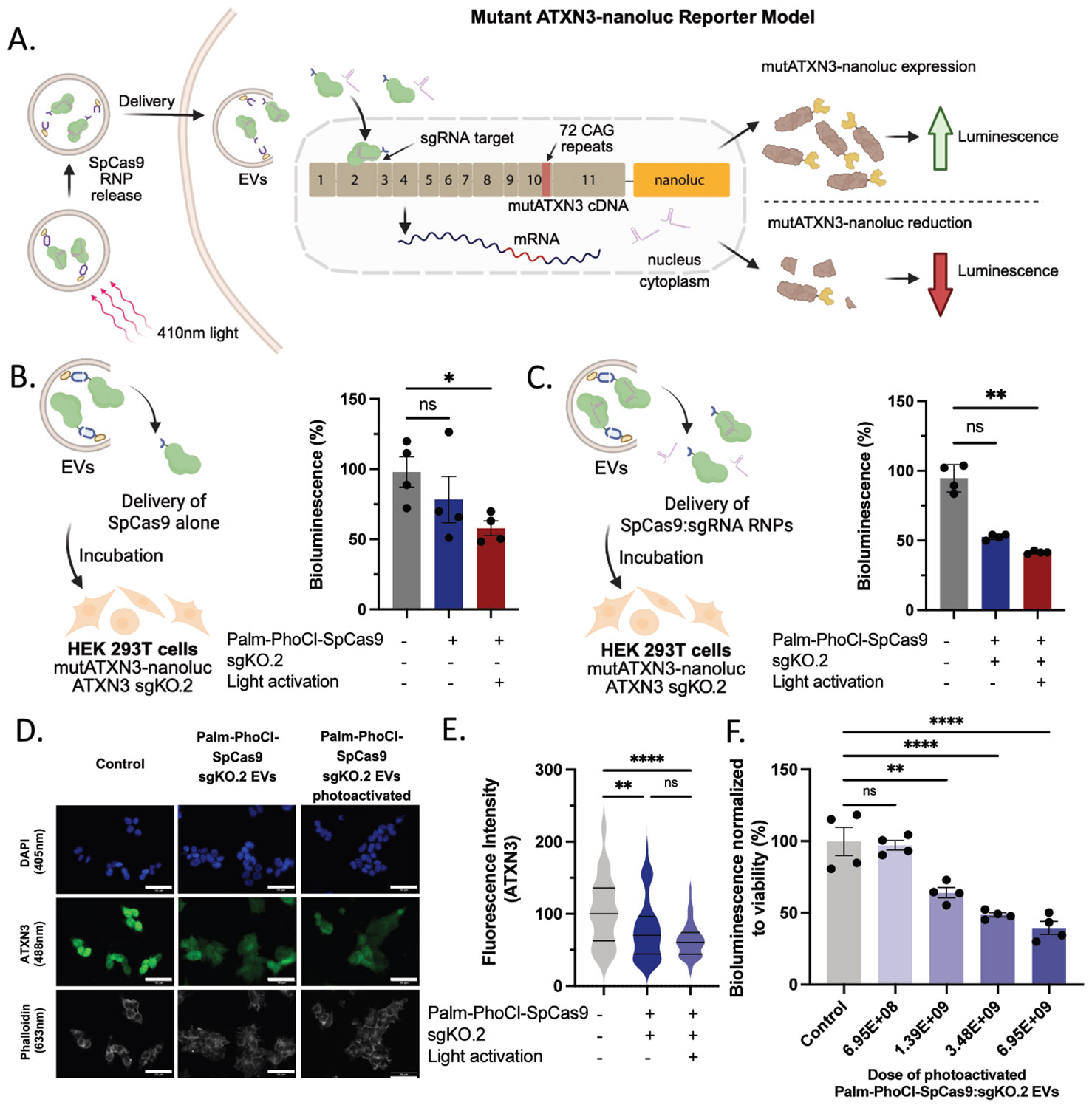
Photoactivated EVs loaded with Palm-PhoCl-SpCas9:sgKO.2 RNPs reduce ATXN3 expression *in vitro*. A. Schematic representation of the reporter system to evaluate EVs delivery of CRISPR-Cas9 *in vitro*. A stable HEK 293T cell line was generated using lentiviral vectors to overexpress nanoluciferase fused to the C-terminal of mutant ATXN3 (mutATXN3-nanoluc) and a sgRNA targeting *ATXN3* (ATXN3 sgKO.2). B. Photoactivated or unexposed EVs loaded with Palm-PhoCl-SpCas9 alone were incubated in HEK 293T cells expressing mutATXN3-nanoluc and *ATXN3* sgKO.2. Bioluminescence was measured after 72 h of incubation (N = 4). Data are expressed as mean ± SEM. Statistical analysis was performed using the Kruskal–Wallis test followed by Dunn’s multiple comparisons post hoc test. *p ≤ 0.05. C. Photoactivated or unexposed EVs loaded with Palm-PhoCl-SpCas9:sgKO.2 were incubated in HEK 293T cells expressing mutATXN3-nanoluc and *ATXN3* sgKO.2. Bioluminescence was measured after 72 h of incubation (N = 4). Data are expressed as mean ± SEM. Statistical analysis was performed using the Kruskal–Wallis test followed by Dunn’s multiple comparisons post hoc test. **p ≤ 0.01. D. Empty EVs (control) and EVs loaded with Palm-PhoCl-SpCas9:sgKO.2 (with and without photoactivation) were incubated for 48 h in HEK 293T cells expressing mutATXN3-nanoluc and *ATXN3* sgKO.2. Cells were passaged for 2 weeks and then collected for analysis. Control and treated cells (with and without photoactivation) were immunolabelled with antibody against ataxin-3 (green), labelled with DAPI (blue) and phalloidin (white) and analyzed via fluorescence microscopy (scale bar represents 50um). E. Ataxin-3 fluorescence intensity from single cells was quantified using Fiji - ImageJ software (N = 60). Data are presented as a violin plot. Statistical analysis was performed using the Kruskal–Wallis test followed by Dunn’s multiple comparisons post hoc test. **p ≤ 0.01; ****p ≤0.0001. F. Photoactivated EVs loaded with Palm-PhoCl-SpCas9:sgKO.2 were incubated in HEK 293T cells expressing mutATXN3-nanoluc and *ATXN3* sgKO.2 in four different doses. Bioluminescence and cell viability were measured after 72 h of incubation (N = 4). Data are expressed as mean ± SEM. Statistical analysis was performed with one-way ANOVA with Dunnett’s post hoc test. **p ≤ 0.01; ****p ≤0.0001.

Next, to evaluate *ATXN3* editing, we firstly designed a luminescence reporter system by fusing the mutant *ATXN3* (mut*ATXN3*) cDNA to nanoluciferase (nanoluc) reporter gene (mut*ATXN3*-nanoluc). The effectiveness of our system was demonstrated by a decrease in luminescence with increasing doses of transfected SpCas9 and sgKO.2 (Supplementary Fig. 7). Once the luminescence reporter system was validated, HEK 293T cells were transduced with lentiviral vectors encoding mut*ATXN3*-nanoluc and sgKO.2, creating a stable cell line expressing mutATXN3 protein and *ATXN3* sgKO.2 (Fig. 4A).

We started by evaluating the knockout efficacy of EV-mediated delivery of SpCas9 protein alone (Fig. 4B). Palm-PhoCl-SpCas9 loaded EVs were photoactivated and incubated in HEK 293T cells expressing mutATXN3-nanoluc and sgKO.2. Palm-PhoCl-SpCas9 EVs exposed to 410 nm light resulted in a significant 42.13% reduction in mutATXN3-nanoluc luminescence relative to empty EVs (control), compared with a 21.74% reduction observed for Palm-PhoCl-SpCas9 EVs not exposed to 410 nm light (Fig. 4B). Next, using the same reporter system, cells were incubated with EVs loaded with Palm-PhoCl-SpCas9:sgKO.2 RNPs, with or without exposure to 410 nm light. Photoactivated Palm-PhoCl-SpCas9:sgKO.2 loaded EVs resulted in a 58.57% reduction of mutATXN3-nanoluc luminescence relative to the control, compared with a 47.49% reduction observed for unexposed Palm-PhoCl-SpCas9: sgKO.2 loaded EVs (Fig. 4C). These results suggest that EVs can effectively deliver active SpCas9 nuclease alone or SpCas9:sgRNA RNPs to recipient cells and that photo-induced release of SpCas9 from the Palm motif improves editing efficiency compared with empty EVs (control). This effect is not associated with fusion of SpCas9 to Palm or Palm-PhoCl, since SpCas9 nuclease activity is not significantly affected (Supplementary Fig. 8). Next, mut*ATXN3*-nanoluc reporter cells incubated with Palm-PhoCl-SpCas9:sgKO.2 loaded EVs were expanded and passaged for two weeks to confirm stable editing across passages (~5 passages). Immunocytochemistry was performed and cells were stained with anti-ATXN3 antibody (1H9), DAPI and phalloidin (Fig. 4D). Quantification of ATXN3 fluorescence confirmed a significant reduction in the levels of ATXN3 after two weeks of EV-mediated delivery of SpCas9:sgKO.2 RNPs, with a 36.45% (mean) reduction of ATXN3 fluorescence with photoactivated Palm-PhoCl-SpCas9:sgKO.2 loaded EVs, compared to 21.10% (mean) reduction with Palm-PhoCl-SpCas9:sgKO.2 loaded EVs unexposed to 410 nm light. This demonstrates a significant sustained knockout mediated by the transient delivery of active SpCas9: sgKO.2 RNPs by EVs (Fig. 4E). We further evaluated the knockout efficiency of the system in a titration study. Photoactivated EVs loaded with Palm-PhoCl-SpCas9:sgKO.2 RNPs were incubated in reporter cells in four incremental doses (6.95 × 10^8^, 1.39 × 10^9^, 3.48 × 10^9^ and 6.95 × 10^9^ particles), resulting in a dose-dependent decrease of mutATXN3-nanoluc luminescence normalized to viability of up to 60.48% (Fig. 4F).

Overall, our results suggest that EVs loaded with Palm-PhoCl-SpCas9:sgKO.2 RNPs were able to significantly reduce ATXN3 in HEK 293T reporter models, highlighting the effect of photo-induced release of SpCas9 to improve delivery efficiency in recipient cells.

### 2.5. Palm-PhoCl-SpCas9:sgKO.2 RNP loaded EVs expressing VSV-G mediate ATXN3 knockout in SCA3 patient-derived iPSCs and animal models

Viral envelope glycoproteins have been reported to improve nano-particles’ cargo delivery and/or endosomal escape in recipient cells [29–31]. To test this hypothesis, we generated hybrid EVs expressing three different viral envelope glycoproteins. We expressed VSV-G, RVG or FuGB2 glycoproteins on the surface of CD63-nanoluc EVs to evaluate internalization in Neuro-2a cells (Supplementary Fig. 9). CD63-nanoluc EVs expressing VSV-G, RVG or FuGB2 were incubated for 12h in Neuro-2a cells. Among VSV-G, RVG or FuGB2 particles, VSV-G resulted in the highest internalization, peaking at 12 h with 2.66-fold increase compared to CD63-nanoluc EVs alone (control), followed by FuGB2 and RVG CD63-nanoluc particles (Supplementary Fig. 9).

**Fig. 5.**
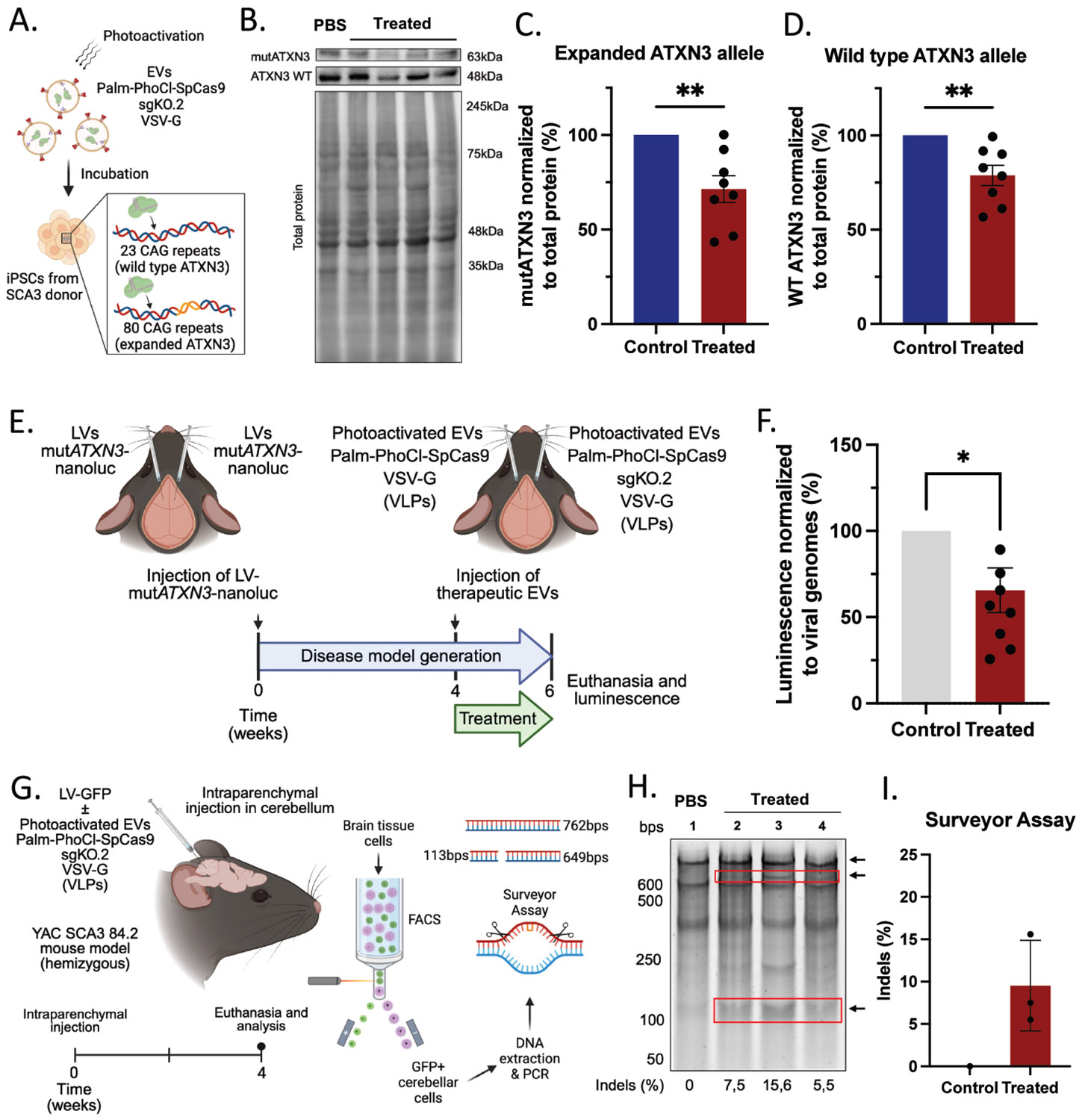
Palm-PhoCl-SpCas9:sgKO.2 RNP EVs expressing VSV-G mediate *ATXN3* knockout in SCA3 patient-derived iPSCs and animal models. A. iPSCs reprogrammed from dermal fibroblasts of SCA3 donors were incubated with photoactivated Palm-PhoCl-SpCas9:sgKO.2 EVs expressing VSV-G. After 72 h, cells were collected to assess the silencing of the wild-type and mutant *ATXN3* alleles. ATXN3 expression was evaluated by Western blot and normalized to total protein levels. B. Representative Western blot of patient-derived iPSCs incubated with photoactivated Palm-PhoCl-SpCas9:sgKO.2 EVs expressing VSV-G. Membranes were stained for total protein and incubated with anti-ATXN3 antibody, for the visualization of expanded (80 CAG repeats) and wild type (23 CAG repeats) ATXN3 alleles. C. Proteins levels of expanded ATXN3 allele (80 CAG repeats) normalized to total protein after SCA3 iPSCs incubation with photoactivated Palm-PhoCl-SpCas9: sgKO.2 EVs expressing VSV-G (N = 5 controls; N = 8 treated). Data are expressed as mean ± SEM. Statistical analysis was performed using a one-sample *t*-test. **p ≤ 0.01. D. Protein levels of wild-type ATXN3 allele (23 CAG repeats) normalized to total protein after SCA3 iPSCs incubation with photoactivated Palm-PhoCl-SpCas9:sgKO.2 EVs expressing VSV-G (N = 5 controls; N = 8 treated). Data are expressed as mean ± SEM. Statistical analysis was performed using a one-sample *t*-test. **p ≤ 0.01. E. Lentiviral vectors encoding mut*ATXN3*-nanoluc were injected in the right and left striata of WT C57BL/6 mice. Four weeks later, EVs were photoactivated *ex-vivo* and injected in the left striatum (EVs loaded with Palm-PhoCl-SpCas9 alone and expressing VSV-G) and in the right striatum (EVs loaded with Palm-PhoCl-SpCas9:sgKO.2 and expressing VSV-G). Two weeks after the injection of EVs, mice were sacrificed and striata lysates were processed by luminescence assay and viral genome quantification. F. Luminescence levels were normalized to viral genomes of the left striatum (control) and right striatum (treated) (N = 8). Data are expressed as mean ± SEM. Statistical analysis was performed using a one-sample *t*-test. *p ≤ 0.05. G. YAC SCA3 84.2 hemizygous mice underwent intraparenchymal injection in the cerebellum with EVs loaded with Palm-PhoCl-SpCas9:sgKO.2 RNPs and expressing VSV-G, photoactivated *ex vivo* and with LVs-GFP. After four weeks, the cerebellum was collected, GFP^+^ cerebellar cells were sorted by fluorescence-activated cell sorting, and Surveyor nuclease assay was performed from the DNA extracted from GFP ^+^ cerebellar cells. H. PAGE gel showing the surveyor nuclease assay results. The two cleavage fragments expected following editing of the *ATXN3* target gene are highlighted. I. Indel levels upon quantification of cleavage fragments on PAGE gel resulting from Surveyor activity in heteroduplexes shows up to 15.6% indels in GFP + cerebellar cells treated with photoactivated Palm-PhoCl-SpCas9:sgKO.2 EVs expressing VSV-G (N = 3 treated). Data are expressed as mean ± SEM.

Given the superior internalization profile of VSV-G expressing EVs, we selected this system to deliver SpCas9:sgRNA RNPs targeting *ATXN3* to iPSCs derived from SCA3 patient fibroblasts. These cells were reprogrammed from dermal fibroblasts of a SCA3 patient encoding a wild type and a mutant *ATXN3* allele with 23 and 80 CAG repeats, respectively (Fig. 5A). Given that our genome editing strategy with SpCas9 is not allele-specific, it is expected to target both the wild-type and expanded *ATXN3*. Photoactivated EVs expressing VSV-G and loaded with Palm-PhoCl-SpCas9:sgKO.2 RNPs were incubated in SCA3 iPSCs for 72 h at a dose of 1.5×10^10^ particles per condition (Fig. 5A). Western blot analysis showed a reduction of 28.58% (mean) and 21.21% (mean) in the levels of mutant and wild-type ATXN3, respectively, when compared to the control (PBS) (Fig. 5B–D), highlighting the delivery efficiency of VSV-G expressing EVs loaded with Palm-PhoCl-SpCas9: sgKO.2 RNPs in SCA3 patient-derived iPSCs. Collectively, these results show the potential of EVs to efficiently deliver SpCas9 RNPs in a SCA3 patient-derived cell model.

We then tested the knockout potential of EVs expressing VSV-G and containing Palm-PhoCl-SpCas9:sgKO.2 RNPs in a SCA3 lentiviral mouse model expressing mut*ATXN3*-nanoluc (Fig. 5E). To generate this model, wild-type C57BL/6 animals underwent intraparenchymal injections in both striata with lentiviral vectors expressing mut*ATXN3*-nanoluc. Four weeks later, the left striatum was injected with VSV-G EVs loaded with Palm-PhoCl-SpCas9 alone (no sgRNA), while the right striatum was injected with VSV-G EVs loaded with Palm-PhoCl-SpCas9:sgKO.2 RNPs at a dose of 5.33×10^9^ particles per injection (Fig. 5E). In both conditions, EVs were photoactivated before injection. The luminescence was assessed in the brains of living animals and in collected brain tissue after two weeks (Supplementary Fig. 10). Levels of luminescence were measured in the dissected striata and normalized for viral genome copies measured by RT-qPCR. We found that normalized luminescence levels were reduced by 34.42% (mean) in the striatum of mice injected with photo-activated VSV-G EVs loaded with Palm-PhoCl-SpCas9:sgKO.2 RNPs when compared with control hemispheres injected with photoactivated VSV-G EVs loaded with Palm-PhoCl-SpCas9 alone (Fig. 5F).

Next, we aimed to evaluate the potential of VSV-G EVs loaded with Palm-PhoCl-SpCas9:sgKO.2 RNPs in a humanized model of SCA3 that expresses endogenous levels of *ATXN3* among different neural cells. For this purpose, we selected the YAC SCA3 84.2 mouse model, which carries two copies of the full human gene of *ATXN3* with 84 CAG repeats, under the control of the human endogenous promoter [32]. To address the gene editing efficiency of our system, we performed intra-parenchymal injections of photoactivated VSV-G EVs loaded with Palm-PhoCl-SpCas9:sgKO.2 RNPs in the cerebellum of hemizygous YAC SCA3 84.2 mice with 12 weeks of age at a dose of 1.43×10^9^ particles per injection (Fig. 5G). Simultaneously, EVs were co-injected with lentiviral vectors encoding GFP reporter to enrich for targeted cells. Four weeks post-injection, the mice were sacrificed, the cerebellum was collected, and GFP + cells were sorted by fluorescence-activated cell sorting. Approximately 1% of GFP + cells were sorted from all events analyzed (Supplementary Fig. 11). Genomic DNA was extracted from GFP + cerebellar cells, and *ATXN3* knockout was evaluated by Surveyor nuclease assay (Fig. 5H), revealing an editing efficiency of up to 15.6 % (Fig. 5I). Collectively, our results demonstrate the potential of EVs for the transient delivery of genome editing machinery, namely SpCas9: sgRNA RNPs in SCA3 patient-derived iPSCs, and in two distinct SCA3 animal models.

## 3. Discussion

In this study, we developed EV-based platforms to deliver SpCas9: sgRNA RNPs targeting exon 2 of *ATXN3* as a novel therapeutic strategy for SCA3, validating this approach in multiple cellular models, patient-derived iPSCs and two animal models.

Firstly, we selected a small palmitoylation (Palm) motif that enhanced the enrichment of SpCas9 protein in EVs. Additionally, we introduced a photocleavable linker between the EV-packaging Palm motif and SpCas9, that is cleaved upon 410 nm light exposure in purified EV preparations, allowing the release of SpCas9 from the EV-packaging Palm. Our results suggest photo-induced release of SpCas9 might facilitate the SpCas9’s re-direction toward the nucleus of the target cell. We demonstrated high packaging efficiencies of SpCas9 protein and sgRNAs in EVs, which were efficiently internalized by recipient cells. SpCas9: sgRNA RNP loaded EVs were able to partially knockout *ATXN3* in reporter models *in vitro*. Additionally, VSV-G co-expression on the surface of the particles improved internalization in a neuronal cell line. Finally, Palm-PhoCl-SpCas9:sgKO.2 loaded EVs expressing VSV-G induced mutATXN3 knockout in iPSCs derived from fibroblast of SCA3 patients by 28.58% and mediated gene editing knockout of *ATXN3* in two different animal models of SCA3.

Over the past years, several strategies have been developed to load CRISPR-Cas9 enzymes in EVs. Endogenous loading of gene editing agents in EVs has been mostly achieved by engineering the tetraspanins CD9, CD63, or CD81 [33–35], the ARRDC1 protein [36], or by attaching SpCas9 to membrane-binding proteins through palmitoylation and/or myristoylation modifications [24,34]. We compared the loading capacity of Palm and tetraspanins CD9 and CD63 by fusing these proteins to the N-terminal of SpCas9 under the regulation of the Ef1a promoter. Palm resulted in higher levels of SpCas9 protein loaded in EVs derived from HEK 293T cells. We selected Palm due to its smaller size, which we hypothesized would minimize its impact on SpCas9 protein structure. Palmitoylation also avoids potential unwanted gain-of-function effects associated with fusion to EV-enriched proteins such as the tetraspanins CD63 and CD9, while remaining a reversible modification that can be dynamically regulated by cellular depalmitoylases. Moreover, myristoylation-palmitoylation tagged SpCas9 EVs have been reported to mediate higher levels of editing than CD9-based SpCas9 loaded EVs in reporter cells [34].

In target cells, SpCas9 must be directed into the nucleus by tagging a nuclear localization signal. Several reports have developed dimerization systems to prevent gene editing enzymes from becoming entrapped in the cytoplasm of target cells or being re-secreted in EVs due to fusion with EV-packaging sequences. Examples include the FRB-FKBP [34,37], split GFP or GFP nanobody approaches [35,38], ARRDC1 and ww-domains [36], or through RNA binding protein systems such as RNA aptamer com-Com ABP [39] and MS2-MCP domains [40–42]. However, several protein-protein or protein-RNA dimerization systems do not effectively control the release of their therapeutic payload to packaging sequences in recipient cells. The FRB-FKBP system can transiently mediate cargo loading into EVs, however it still requires the addition of dimerization molecules to cell cultures. We reasoned that using proteins with optogenetic properties would enable light-induced control of cargo loading or release from EVs. The light (488 nm)-mediated interaction of CRY2-CIBN has been harnessed for the loading of cargo into EVs, yet this system requires prolonged light exposure of cell cultures [34]. For this reason, we selected photocleavable PhoCl protein [28] which, when placed between the Palm and SpCas9 coding sequences, mediated their dissociation upon ~400 nm light exposure of purified EVs *ex vivo*, after their isolation. We observed that increasing the exposure time of SpCas9-loaded EVs to 410 nm light increased its release from the Palm-PhoCl protein.

We then aimed at maximizing the loading efficiency of sgRNAs in EVs. We demonstrated that overexpression of sgKO.2 in producer cells together with Palm-PhoCl-SpCas9 results in high sgRNAs loading in EVs. This finding suggests sgRNAs may bind to SpCas9 in the cytoplasm, forming the RNP complex upon loading into EVs, as previously reported [35,43]. The formation of a SpCas9:sgRNA RNP complex confers both protection and indirect EV-packaging properties to the sgRNA. This observation poses an advantage for developing SpCas9 loaded EVs for several applications. Although sgRNAs do not have a natural packaging ability, we were able to detect sgRNA in EVs derived from cells overexpressing sgKO.2. Active loading of sgRNA in EVs generally requires modification of the sgRNA sequence to mediate binding of RNA-binding motif to EV packaging proteins [42,44], which could hinder its target engagement or increase risk of potential off-targets.

As proof of concept of therapeutic application, we targeted *ATXN3* gene associated with Spinocerebellar Ataxia Type 3 (SCA3). The overexpansion of CAG triplet repeats in the exon 10 of *ATXN3* is responsible for the development of SCA3, a progressive neurodegenerative disorder for which there are no disease-modifying treatments available. The expanded polyglutamine tract in mutant ATXN3 protein is responsible for protein aggregation and neuronal death in the brain [2]. To inactivate *ATXN3* expression and halt the accumulation and aggregation of toxic mutant ATXN3 protein, we used a previously designed sgRNA to promote a DSB in exon 2 of the *ATXN3* gene (sgKO.2) [25]. This targeted disruption causes a frameshift mutation leading to permanent inactivation of *ATXN3* expression. While inactivation of *ATXN3* gene also results in a loss-of-function of the wild type *ATXN3*, so far, *ATXN3* knockout animals have been shown to remain viable and fertile with no reduction in lifespan [45]. *ATXN3* knockout models present minor behavioral changes [46]. We demonstrated that SpCas9:sgRNA EVs reduced ATXN3 expression by up to 60.48% in reporter models *in vitro*. Although inactivation of the *ATXN3* gene may mitigate downstream toxic events driven by the mutant ATXN3 protein, it is unlikely to eliminate toxicity arising from expanded CAG repeat mRNA or from Repeat-Associated Non-AUG (RAN) translation-mediated production of toxic dipeptide repeat proteins (DPRs).

To improve the delivery efficiency of therapeutic cargos to target cells, several reports have utilized viral envelope proteins, such as VSV-G, RVG and others, to pseudotype cell-derived vesicles [29–31]. Indeed, VSV-G has been extensively used to promote cell entry and endosomal escape, resulting in high editing efficiency in *in vitro* models. We hypothesized that co-expression of viral envelope proteins might improve the internalization of SpCas9:sgRNA RNP loaded EVs in more relevant SCA3 cellular and animal models. Indeed, we found that expression of VSV-G in EVs incubated into a neuronal cell line improves the internalization of CD63-nanoluc EVs by 2.66-fold, compared to RVG and FuGB2. Given that neurons are the most affected cell type in SCA3 pathology [2], we introduced the VSV-G targeting moiety on the surface of Palm-PhoCl-SpCas9:sgKO.2 loaded EVs. EVs coated with VSV-G and loaded with Palm-PhoCl-SpCas9:sgKO.2 RNPs were able to reduce mutATXN3 protein expression by 28.58% in SCA3 patient-derived iPSCs. Although the exact mechanism by which VSV-G improves cellular delivery is not fully understood, evidence suggests it contributes to endosomal escape from endosomes, preventing the degradation of internalized particles and their content by lysosomes [38,47]. Although expression of viral envelope proteins may trigger immune responses upon systemic delivery *in vivo* [48], injecting the lentiviral vector into the CNS was found not to elicit an immune response against any of the lentiviral proteins, including VSV-G [48], highlighting the potential of VSV-G expressing particles as a promising strategy for gene therapy.

Lastly, we demonstrated the efficacy of Palm-PhoCl-SpCas9:sgKO.2 loaded EVs expressing VSV-G in two different animal models of SCA3. First, we established a luciferase reporter lentiviral model by injecting LVs encoding mut*ATXN3*-nanoluc into the striata of wild-type C57BL/6 mice. Luciferase-based reporter animal models have been previously reported by us and others, allowing for continuous monitoring of therapeutic efficacy in living animals [37,49]. The disease model was established after four weeks of LVs injection, allowing stable expression of the mut*ATXN3*-nanoluc transgene. Photoactivated Palm-PhoCl-SpCas9:sgKO.2 loaded EVs expressing VSV-G reduced the normalized luminescence levels by 34.42% relative to photoactivated VSV-G EVs loaded with Palm-PhoCl-SpCas9 alone. We then demonstrated that our engineered EVs mediate genome editing in the YAC SCA3 84.2 mouse model, which carries the full human *ATXN3* gene with 84 CAG repeats. Upon intraparenchymal injection in the cerebellum, photoactivated Palm-PhoCl-SpCas9:sgKO.2 loaded EVs coated with VSV-G and co-injected with lentiviral vectors encoding GFP mediated genome editing in GFP + cerebellar cells, resulting in up to 15.6 % indels at the target site. Due to the limited distribution of EVs in the brain following intraparenchymal delivery, reaching other brain regions with this strategy would require multiple injections or alternative administration routes. Intracerebroventricular administration has been previously shown to be an efficient strategy to deliver EVs to the CNS [50]. Scaling up the production and functionalization of EVs for broad CNS delivery through less invasive routes, such as intravenous or intranasal delivery may enable phenotype amelioration in multiple SCA3 animal models [49,51].

These data suggest that transient delivery of SpCas9:sgRNA loaded, VSV-G expressing EVs enables partial *ATXN3* knockout in animal models, providing a proof of concept for *in vivo* therapeutic efficacy in the context of SCA3.

Overall, this work provides evidence that this EV-based platform enables transient delivery of CRISPR-Cas9 tools *in vitro* and *in vivo*, by systematically addressing: 1) EV packaging of SpCas9 and sgRNA in the producer cell; 2) release and nucleus redirection of SpCas9 in recipient cells; 3) VSV-G expression to improve endosomal escape; and 4) partial knockout of *ATXN3* expression in SCA3 patient-derived iPSCs and two SCA3 animal models. This study provides proof of concept for a therapeutic strategy for SCA3 that may be applicable to other monogenic brain disorders.

## 4. Materials and methods

### 4.1. Plasmid cloning

For the cloning of CD63-SpCas9 and CD9-SpCas9 plasmids, CD63 and CD9 primers were designed to introduce 20-25 nucleotide homology regions to the backbone SpCas9 vector. CD9 and CD63 were then amplified by PCR from a plasmid template and the amplicons purified. SpCas9 vector was linearized using the restriction enzymes BshT1 and Eco47VIII. CD63 and CD9 inserts were assembled into the vector using the NEBuilder HiFi DNA Assembly mix, according to the manufacturer’s instructions. Palmitoylation motif MLCCMRRTKQ was generated by designing overlapping oligos and direct cloning into the overhangs of the digested SpCas9 vector.

### 4.2. Cell culture and transfection

Human embryonic kidney 293 cells stably expressing the SV40 large T antigen (HEK 293T) were cultured in Dulbecco’s Modified Eagle Medium (DMEM) high glucose (Gibco), supplemented with 10 % fetal bovine serum (FBS, Biowest) and 1 % Penicillin/Streptomycin (P/S, Life Technologies) in an incubator at 37 °C and 5% CO_2_. To produce EVs or LVs, cells were plated at 50% confluency, transfected 24 h later and culture media changed 6h following transfection. Conditioned media was collected 48-72 h later and processed.

### 4.3. Isolation of extracellular vesicles (EVs)

EVs were isolated using a differential ultracentrifugation protocol. Conditioned media from HEK 293T cells was collected 48-72 h post-transfection and centrifuged at 300 g for 15 min to remove cell debris. The supernatant was then centrifuged at 16,500 g for 1 h at 4 °C to remove larger vesicles using a SW28 rotor (Beckman Coulter). The resulting supernatant was filtered through a 0.22 μm membrane (Syringe filter PES 0.22um SFPE-22E-050 or Milipore Stericup PVDF 0.22um) and further centrifuged at 100,000 g for 2 h at 4 °C to pellet EVs (using a SW28 rotor from Beckman Coulter). A washing step in PBS is undertaken at 100,000 g for 2 h at 4 °C. The pelleted EVs were resuspended in ice-cold PBS. For functional assays, EVs were stored at 4 °C for up to 7 days.

### 4.4. Isolation of EVs by density gradient ultracentrifugation

EVs previously isolated by differential ultracentrifugation, as described above, were further purified by density gradient ultracentrifugation using iodixanol. Four layers of iodixanol solutions (40%, 20%, 10%, and 5%) were prepared in 13.2 mL round-bottom ultracentrifuge tubes to a total gradient volume of 9 mL. Following gradient preparation, 1 mL of EVs obtained by differential ultracentrifugation was carefully layered on top of the gradient, resulting in a final volume of 10 mL per tube.

Density gradients were centrifuged at 100,000 g for 18 h at 4 °C using an SW 41 Ti swinging-bucket rotor (Beckman Coulter). After centrifugation, ten 1 mL fractions were collected sequentially from the top of the gradient. Each fraction was diluted in PBS and ultracentrifuged at 100,000 g for 2 h to remove iodixanol. The resulting EV pellets were resuspended in PBS and characterized by western blotting and nanoparticle tracking analysis (NTA).

### 4.5. Transmission electron microscopy (TEM)

EVs obtained via differential ultracentrifugation were treated with 2 % PFA/PBS for fixation and then allowed to adhere to Formvar-carbon coated grids (TAAB Laboratories) for 5 min. Any surplus liquid was carefully removed from the grid surface using filter paper (Whatman). The grids were then contrasted with 2 % uranyl acetate for 1 min, and the excess stain was blotted away, before allowing the sample to air dry. Observations were conducted using a Tecnai G2 Spirit BioTwin electron microscope (FEI) at 100 kV.

### 4.6. Nanoparticle tracking analysis (NTA)

EV concentration and size were assessed using NTA Version 2.2 Build 0375 instrument (NanoSight NS300 instrument, Malvern Instruments). EVs diluted in PBS were analyzed at a follow rate of 40 arbitrary units (AU), with the acquisition of five videos of 30 s. The number of particles (30-1000 nm) was determined using NTA Software 2.2. Samples were diluted to achieve an average of 20 to 100 particles per frame, following the manufacturer’s instructions.

### 4.7. Western blotting

Cells and EVs were collected in RIPA buffer [50 mM Tris-base (Thermo Scientific); 150 mM sodium chloride (NaCl, Acros Organics); 5 mM ethylene glycol tetraacetic acid (EGTA, Thermo Scientific); 1 % Triton X-100 (Thermo Scientific); 0.5 % sodium deoxycholate (Sigma-Aldrich); 0.1 % sodium dodecyl sulfate (SDS, Thermo Scientific); pH = 7.5], supplemented with a protease inhibitor cocktail (Roche) and 200 μM phenylmethylsulfonyl fluoride (PMSF, Sigma-Aldrich), 1 mM dithiothreitol (DTT, Thermo Scientific), 1 mM activated sodium orthovanadate (Sigma-Aldrich) and 5 mM sodium fluoride (NaF, Acros Organics). Protein concentration of cell lysates was determined using Bradford assay (Bio-Rad Laboratories) according to the manufacturer’s instructions. The protein concentration of EVs was measured using the Micro BCA^™^ Protein Assay Kit (ThermoFisher Scientific). Samples were denatured at 95 °C for 5 min with 6x sample buffer [0.375 M Tris pH 6.8 (Sigma-Aldrich), 12 % SDS (Sigma-Aldrich), 60 % glycerol (Sigma-Aldrich), 0.6 M DTT (Sigma-Aldrich) and 0.06 % bromophenol blue (Sigma-Aldrich)]. For iPSCs, 50 μg of protein were loaded, otherwise 10-20 μg of protein from cell lysates were used. For EVs, 5-15 μg of protein were loaded. For SpCas9 standard curve, purified SpCas9 was loaded (Alt-R^™^ SpCas9, IDT). Samples were resolved by Sodium dodecylsulfate polyacrylamide gel electrophoresis (SDS-PAGE) on 8 %, 10 %, or 12 % gels depending on the target protein. Samples were transferred onto polyvinylidene fluoride (PVDF) membranes (GE Healthcare). Total protein labeling was performed using No-Stain^™^ Protein Labeling Reagent (ThermoFisher Scientific) according to the manufacturer’s instructions. Membranes were blocked by incubation in 5 % non-fat milk powder in 0.1 % Tween 20 (VWR Chemicals) in Tris-buffered saline (TBS-T), for 2 h and incubated overnight at 4 °C with primary antibodies (Alix - BD Biosciences, 611620, 1:1000; calnexin - Santa Cruz, sc-11397, 1:1000; CRISPR-SpCas9 - abcam, ab189380, 1:5000; flotillin-1 - BD Biosciences, 610820, 1:1000; HSC70 - GeneTex, GTX101144, 1:1000; and ataxin-3 - Millipore, MAB5360 1:1000). Membranes were washed in TBS-T for 10 min, three times, and incubated with an alkaline phosphatase-linked secondary goat anti-mouse/anti-rabbit antibody (1:10,000; Thermo Scientific Pierce) at room temperature for 1 h. Visualization of bands was carried out using Enhanced Chemifluorescence substrate (ECF, GE Healthcare) in a chemifluorescence imaging system (ChemiDoc Imaging System, Bio-Rad). Analysis and quantification were carried out based on the optical density of scanned membranes in ImageLab version 5.2.1 (Bio-Rad Laboratories) or Fiji - ImageJ software.

### 4.8. Photo-induced PhoCl cleavage and imaging

For confocal imaging, HEK 293T cells expressing Palm-PhoCl-SpCas9 were exposed to 405 nm laser using a Zeiss Axio Imager Z2 and Zeiss LSM 510 Meta confocal microscope (Carl Zeiss MicroImaging), equipped with Plan-Apochromat 63x/1.40 Oil DIC M27 (420782-9900) and analyzed in ZEN Image software. Images were captured with 488/507 nm and 587/610 nm. Otherwise, ZOE Fluorescent Cell Imaging System blue channel (355/40 nm) was used for photo-induced cleavage of PhoCl and imaging with Green Channel (480/517) and Red Channel (556/615) was performed.

For EVs loaded with Palm-PhoCl-SpCas9, photoconversion was performed with an in-house 410 nm light laser. Unless otherwise specified, 410 nm light exposure was conducted for 5 min.

### 4.9. Amplicon sequencing and data analysis

Genomic DNA was harvested approximately 72 h after transfection by removing culture medium and lysing cells in 100 μl of quick lysis buffer (20 mM HEPES, pH 7.5; 100 mM KCl; 5 mM MgCl_2_; 5% glycerol; 25 mM DTT; 0.1% Triton X-100; and 60 ng μl^−1^ proteinase K (NEB)). Lysates were incubated at 66 °C for 6 min followed by heat inactivation at 95 °C for 2 min. Genomic DNA was purified using a 0.8x ratio of paramagnetic beads prepared as previously described [52].

Editing efficiencies were quantified by targeted amplicon sequencing using a two-step PCR-based Illumina library preparation workflow. In the first PCR (PCR-1), target genomic loci were amplified from ~60 ng genomic DNA using Q5 High-Fidelity DNA Polymerase (NEB) and locus-specific primers (Supplementary Table 2) with the following cycling conditions: 98 °C for 2 min; 35 cycles of 98 °C for 10 s, 63-66 °C for 10 s (exact annealing temperature described in Supplementary Table 2), and 72 °C for 20 s; followed by a final extension at 72 °C for 1 min. PCR-1 products were purified using paramagnetic beads at a 1.8x ratio.

For the second PCR (PCR-2), ~25 ng of purified PCR-1 product was amplified to append Illumina adaptor and barcode sequences using Q5 polymerase and primers previously described [53]. Cycling conditions were: 98 °C for 2 min; 10 cycles of 98 °C for 10 s, 65 °C for 30 s, and 72 °C for 30 s; followed by a final extension at 72 °C for 5 min. Libraries were pooled, quantified using a Qubit dsDNA High Sensitivity assay (Thermo Fisher), and sequenced on an Illumina MiSeq using a 300-cycle v2 kit. On-target editing efficiencies were quantified using CRISPResso2 run in standard mode, with indel frequencies reported as the percentage of modified reads from the CRISPResso_quantification_of_editing_frequency.txt output.

### 4.10. Assessment of off-target editing

Putative off-target sites for *ATXN3* sgKO.2 were predicted using Cas-OFFinder with parameters of ≤3 mismatches, with the following spacer: GTGCCTGAATAACTTATTGCA and NGG PAM, with no RNA or DNA bulges (Supplementary Table 2). Only 4 predicted off-target loci were nominated via these parameters and were therefore selected for experimental validation. Amplicon-specific primers were designed to generate products of 150-250 bp, with the predicted off-target sequence centered within the amplicon and annealing temperatures between 61 °C and 66 °C. PCR-1 primers were generated by appending Illumina adaptor sequences to gene-specific primers.

Cells were transfected with 29 ng SpCas9 nuclease and 13 ng sgKO.2 plasmids, while control samples received SpCas9 nuclease alone. Genomic DNA was collected 72 h post-transfection, and off-target loci were amplified, processed into sequencing libraries, and sequenced as described above. Off-target editing frequencies were quantified using CRISPResso2 in standard mode, and indel rates were calculated as the percentage of modified reads.

### 4.11. Immunocytochemistry

Cells were washed in PBS, fixed with 4 % paraformaldehyde (PFA, Acros Organics) diluted in PBS for 15 min, and permeabilized in PBS with 0.1% Triton X-100 (Sigma-Aldrich) for 10 min. Blocking was performed in PBS with 0.1% Triton X-100 and 3% bovine serum albumin (BSA) (Acros Organics) for 1 h and cells were incubated with primary antibodies in blocking solution at 4 °C overnight (ataxin-3 - Millipore, MAB5360 1:250; ANTI-FLAG M2 - Sigma, F1804, 1:250). Cells were washed three times with PBS for 10 min each and then incubated for 2 h at room temperature with secondary antibodies (Alexa Fluor 488, goat anti-mouse IgG, 1:250, #A11001; Alexa Fluor 568, goat anti-mouse, 1:250, #A11004). Cells were washed three times with PBS for 10 min each and incubated for 5 min with 4′,6-Diamidine-2′-phenylindole dihydrochloride DAPI (1:5000 - Sigma) and Phalloidin (#65906 Sigma). Samples were washed twice in PBS, rinsed with water, and mounted in mounting medium (Dako) on gelatin-coated slides. Imaging was performed in a Zeiss Axio Imager Z2 and Zeiss LSM 510 Meta confocal microscope (Carl Zeiss MicroImaging), quipped with Plan-Apochromat 63x/1.40 Oil DIC M27 (420782-9900) and analyzed in ZEN Image software.

### 4.12. iPSCs culture

Human iPSCs were generated by reprogramming dermal fibroblasts from a SCA3 patient (encoding 23 and 80 CAGs within the wild-type and mutant *ATXN3* alleles respectively) using a non-integrating approach through the delivery of a cocktail consisted of Yamanaka episomal vectors, as previously described [54]. iPSCs were cultured as monolayers in mTeSR Plus medium (STEMCELL Technologies, Catalog # 100-0276) supplemented with 0.5% of Penicillin/Streptomycin on Matrigel-coated (Corning, Ref.354277) 6-well plates. Matrigel was diluted at 1:100 in DMEM/F12 and incubated at 37 °C for 30 min prior to seeding. Cells were maintained at 37 °C in a humidified incubator with 5% CO_2_, with medium replaced every two days. iPSCs were passaged every 5-6 days using Versene (Gibco Ref. 15040066), a non-enzymatic dissociation reagent.

### 4.13. DNA and RNA extraction

DNA and RNA were extracted from cells and brain tissue following the protocol recommendations of the GeneJET Genomic DNA Purification Kit (ThermoFisher Scientific) and RNeasy Plus Micro Kit (Qiagen), respectively. RNA from EV samples was extracted following the protocol recommendation of the Total RNA Purification Maxi Kit (Norgen). DNA and RNA concentration and purity were determined with a NanoDrop^™^ 2000 (ThermoFisher Scientific) and stored at −20 °C and −80 °C, respectively.

### 4.14. cDNA synthesis and RT-PCR

cDNA synthesis was performed following the manufacturer’s instructions of the iScript^™^ cDNA Synthesis Kit (Biorad) and stored at −20 °C. RT-qPCR was performed in real-time quantitative PCR with the SsoAdvanced^™^ SYBR Green Supermix Kit (Biorad) using the following primers: FRW_sgRNA: CTTATTGCAGTTTTAGAGCTAGAAATAGC; REV_sgRNA: CTTGAAAAAGTGGCACCGAGT; FRW_GAPDH: TGTTCGA ACAGTCAGCCGCATCTTC; REV_GAPDH: CAGAGTTAAAAGCAGCCCT GGTGAC).

### 4.15. Lentiviral production

Lentiviral vectors encoding for mutATXN3-nanoluc and sgRNA KO.2 were produced in HEK 293T using a second-generation packaging system, as previously described [55], with slight modifications. Briefly, cells were seeded in 100 mm petri dishes and transfected 24 h later with a 3-plasmid system using polyethylenimine (PEI) linear MW 40000 (Polysciences). Six hours following transfection, culture media was removed, cells were washed in PBS and incubated in fresh culture DMEM high glucose (Gibco) supplemented with 10% FBS (Biowest), 1% Pen/Strep (Life Technologies). Culture media was collected 48-72 h later, filtered with a 450 nm filtering unit (Milipore Stericup PVDF 0.45 μm) and centrifuged at 70,000g for 1.5 h in Optima XPN-100 Ultracentrifuge (Beckman Coulter). For *in vivo* studies, the pellet re-suspended in 1% PBS/BSA (Merck Millipore) was further concentrated at 70,000g for 1.5 h. Viral particles were quantified by assessing HIV-1 p24 antigen levels by ELISA 2.0 (RETRO-TEK, ZeptoMetrix, 0801002), according with the manufacturer’s instructions. Concentrated lentiviral stocks were stored at −80 °C.

### 4.16. Animals

Animal experimental procedures followed the guidelines set by the European Union Directive 86/609/EEC regarding the care and utilization of laboratory animals. This study is a component of a research project that obtained approval from the Center for Neuroscience and Cell Biology ethics committee (ORBEA_66_2015_/22062015 and ORBEA_289), as well as the Portuguese Authority responsible for overseeing animal experimentation, Direcção Geral da Agricultura e Veterinária (DGAV 0421/000/000/2015).

Researchers underwent comprehensive training, including a certification from the Federation of European Laboratory Animal Science Associations (FELASA)-certified course, and were authorized by Portuguese authorities (Direcção Geral de Alimentação e Veterinária) to conduct the experiments. SCA3 YAC84.2 and C57BL/6 mice (obtained from Jackson laboratories and Charles River Laboratories, respectively) were housed in conditions with *ad libitum* access to food and water, following a 12-h light/dark cycle.

Male and female mice, aged between 10 and 12 weeks, were randomly assigned to experimental groups.

### 4.17. Stereotaxic injection in the brain

Wild-type (WT) C57BL/6J or CBA-Tg(ATXN3*)84.2 mice at 10-12 weeks of age were anesthetized by intraperitoneal injection of a mixture of ketamine (75 mg/kg, Nimatek, Dechra) with medetomidine (0.75 mg/kg, DOMTOR, Esteve). Mice were injected into: the striatum (coordinates anteroposterior: 0.6 mm, lateral: ±1.8 mm, ventral: 3.3 mm relative to Bregma) with concentrated lentiviral vectors in a final volume of 2 μL/injection or with EVs in a final volume of 4 μL/injection; in the cerebellum (anteroposterior: 6.0 mm, lateral: 0.0 mm, ventral: 2.9 mm relative to bregma, with Bregma and Lambda aligned) with concentrated EVs and LVs in a final volume of 4 μL/injection. The infusion was performed at a rate of 0.25 μL/min using a 10 μL Hamilton syringe attached to point style 2 needle (Hamilton Company). Five minutes after the infusion was complete, the needle was retracted 0.3 mm and held in place for an additional 3 min before its complete removal from the mouse’s brain. The skin incision was sutured using a 6-0 Prolene^®^ suture (Ethicon, Johnson and Johnson, Brussels, Belgium).

### 4.18. Bioluminescence analysis

Bioluminescence was measured *in vitro* using Furimazine (Nano-Glo, Promega) diluted 1:500 in PBS, and measured according to the manufacturer’s instructions in the LUMIstar Omega (BMG LABTECH). *In vivo*, lentiviral transduction in the brain was monitored by assessing nano-luciferase bioluminescence using IVIS 200 Imaging System (PerkinElmer). Mice were anesthetized by intraperitoneal injection of a mixture of ketamine (75 mg/kg) with medetomidine (0.75 mg/kg). For each measurement, mice were intravenously injected with 100uL of furimazine (1 volume of substrate in 50 volumes of PBS, Nano-Glo Luciferase Assay System - Promega). Bioluminescence images were acquired 5 min after substrate injection. Analysis was performed using Living Image software version 4.3.1 (PerkinElmer).

### 4.19. Cerebellar cell sorting

After 4 weeks post-injection, mice were sacrificed and cerebellum collected. Cerebella were dissected and dissociated with trypsin (0.01%, Sigma, T0303) for 15 min (agitation each 5 min) at 37 °C with DNase (0.045 mg/mL, Sigma, D5025) in Mg2+-free Krebs buffer (120 mM NaCl, 5 mM KCl, 1.2 mM KH_2_PO_4_,13 mM glucose, 15 mM HEPES, 0.3% BSA [pH 7.4]).

Cerebella were then washed with Krebs buffer (with Mg2+) containing FBS (Biowest) to inhibit trypsin activity. Cells were dissociated in this solution and centrifuged. Pellet was resuspended first with a pipet tip, followed by a 1 mL syringe (BD Plastipak^™^) with a needle of 21G (BD Microlance^™^ 3), then filtered through a strainer of 40 *μ* m (Fisherbrand), resuspended in PBS to a round bottom tube and keep on ice until fluorescence-activated cell sorting. Dissociated cerebellar cells were sorted by BD FACSAria (BD Biosciences).

## Supplementary Material

1

2

3

4

5

6

7

8

9

10

11

12

## Data Availability

Data will be made available on request.

## Appendix A. Supplementary data

Supplementary data to this article can be found online at https://doi.org/10.1016/j.biomaterials.2026.124119.
