# Supplementary figures and images for: Extracellular vesicles-mediated delivery of SpCas9 RNPs for therapeutic gene editing in Spinocerebellar Ataxia Type 3
Source: Biomaterials. Author manuscript; Available in PMC 2026 Jun 23. (PMC13288182; doi:10.1016/j.biomaterials.2026.124119)

A.

### sgRNA screening for *ATXN3* exon 2

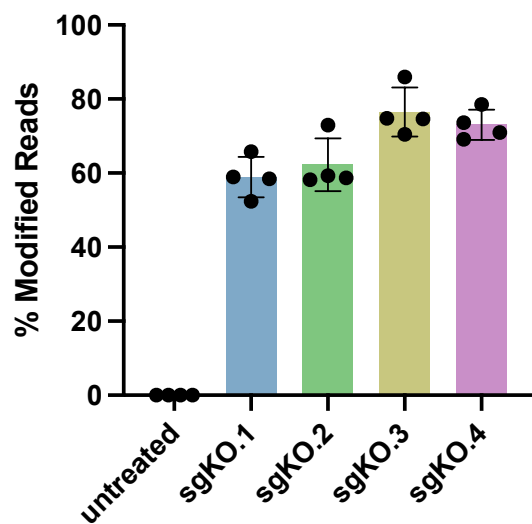

B.

|        | Palm- |   | CD63- |   | CD9- |   |
|--------|-------|---|-------|---|------|---|
| SpCas9 | +     | + | +     | + | +    | + |
| sgKO.2 | -     | + | -     | + | -    | + |

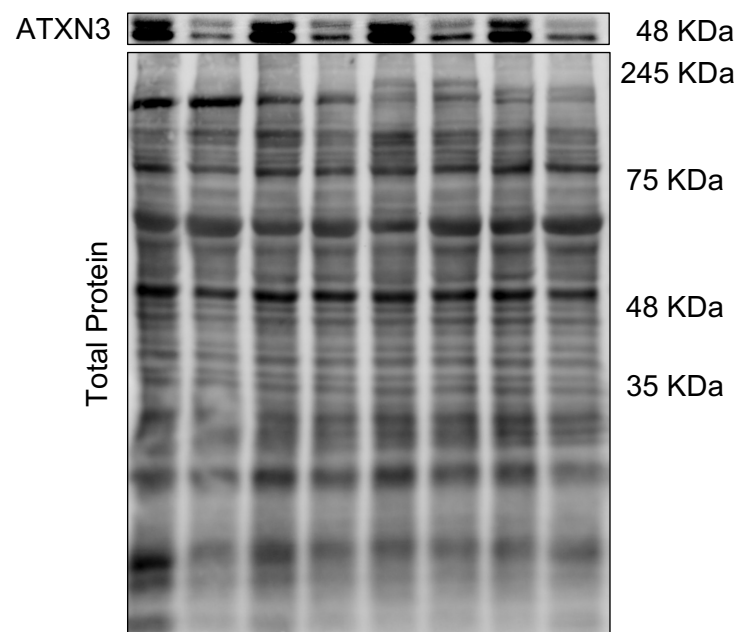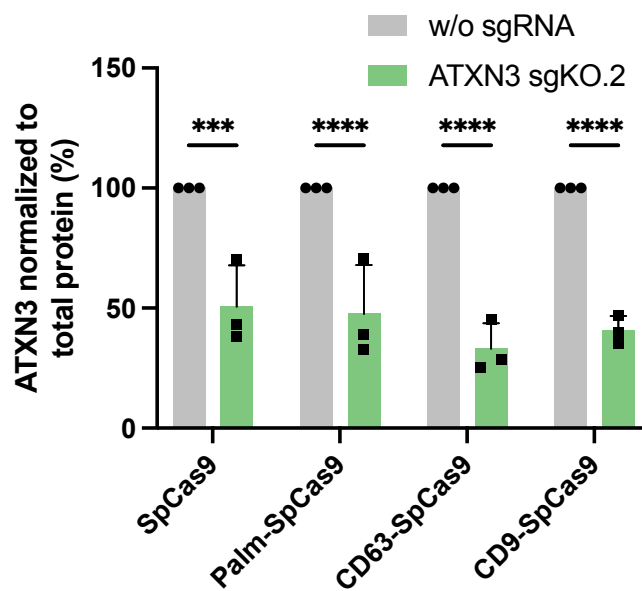

Supplement: 1 [file NIHMS2179600-supplement-1.pdf]

A.

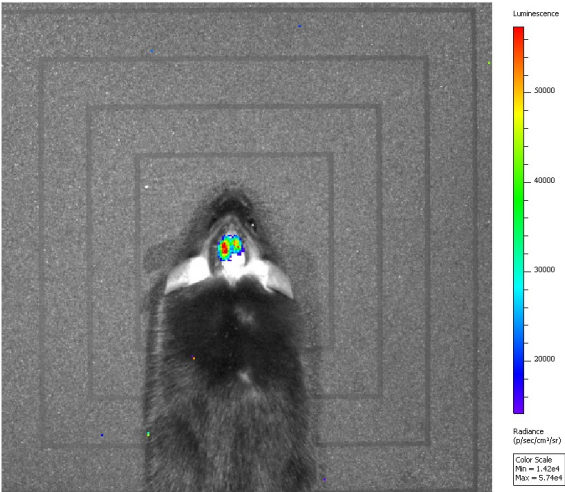

B.

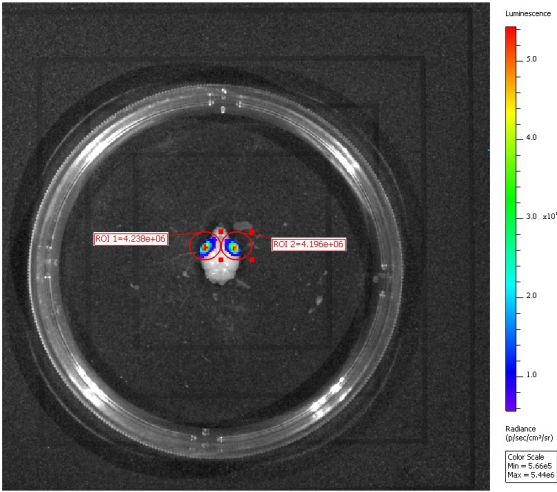

Supplement: 2 [file NIHMS2179600-supplement-2.pdf]

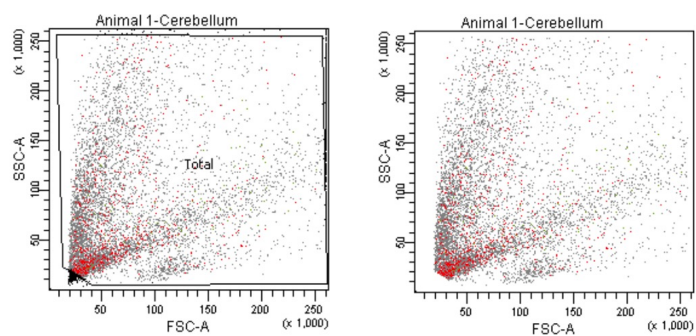

Tube: Cerebellum

| Population | #Events | %Parent | %Total |
|------------|---------|---------|--------|
| All Events | 10,000  | ###     | 100.0  |
| Total      | 7,303   | 73.0    | 73.0   |
| GFP POS    | 71      | 1.0     | 0.7    |
| GFP NEG    | 599     | 8.2     | 6.0    |

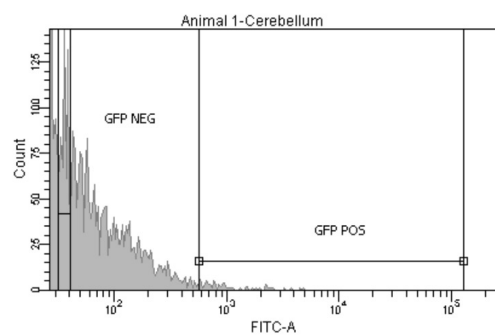

Supplement: 3 [file NIHMS2179600-supplement-3.pdf]

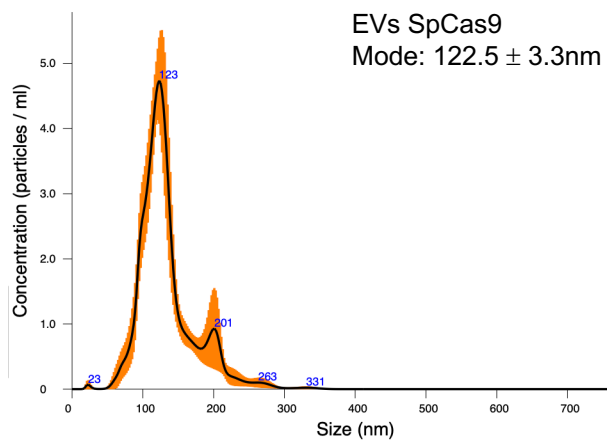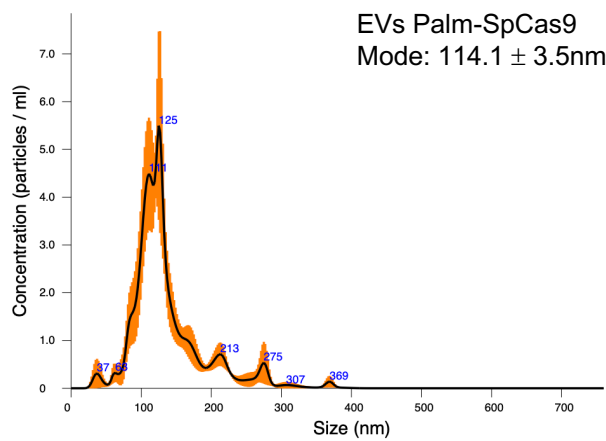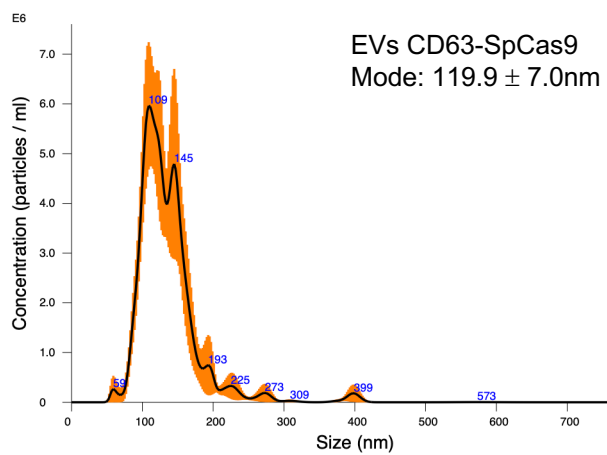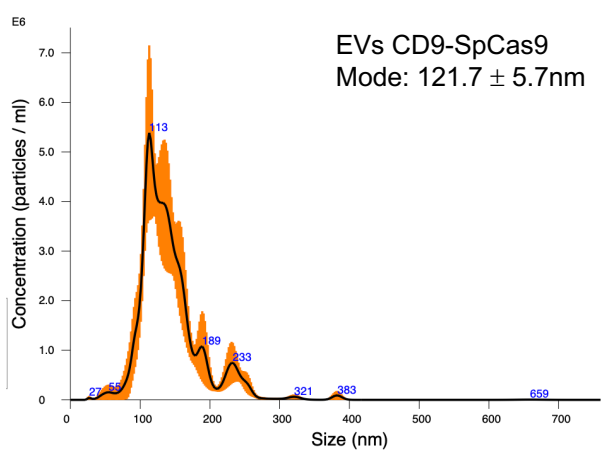

Supplement: 5 [file NIHMS2179600-supplement-5.pdf]

Time after  
exposure

0 seconds

3 seconds

6 seconds

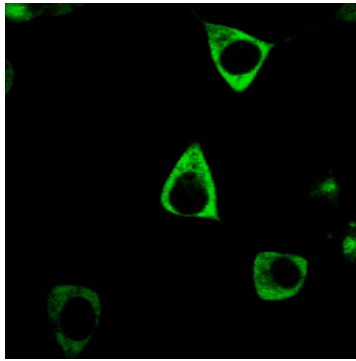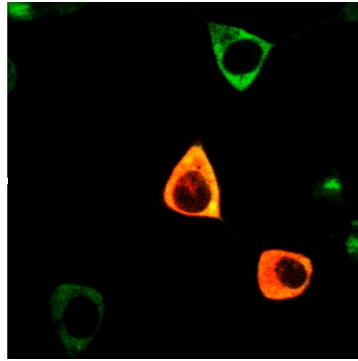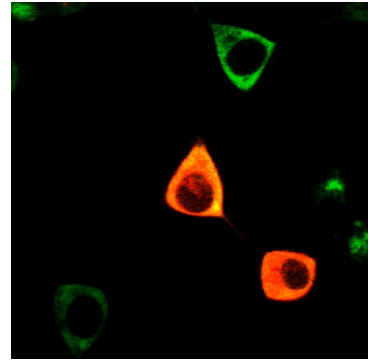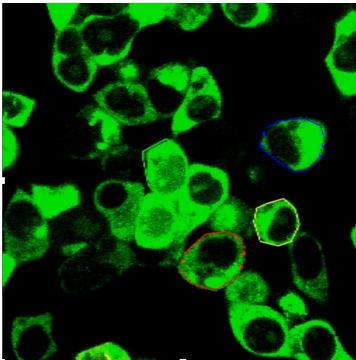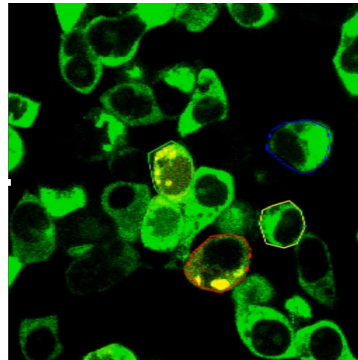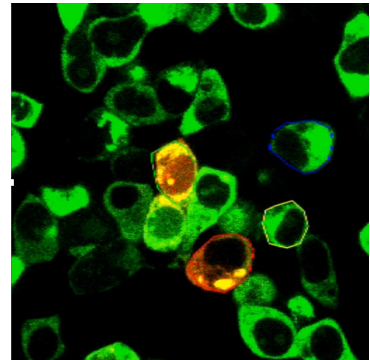

Supplement: 6 [file NIHMS2179600-supplement-6.pdf]

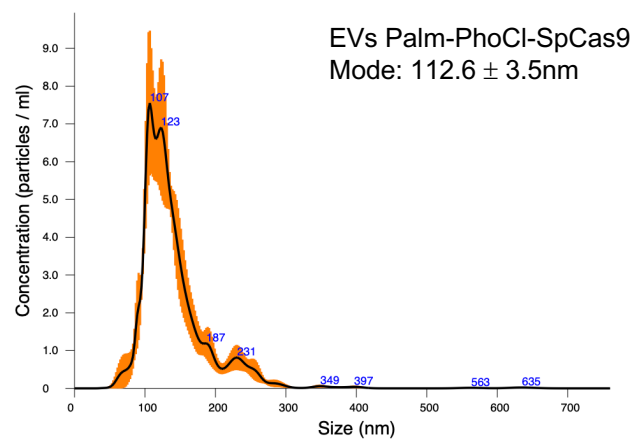

Supplement: 7 [file NIHMS2179600-supplement-7.pdf]

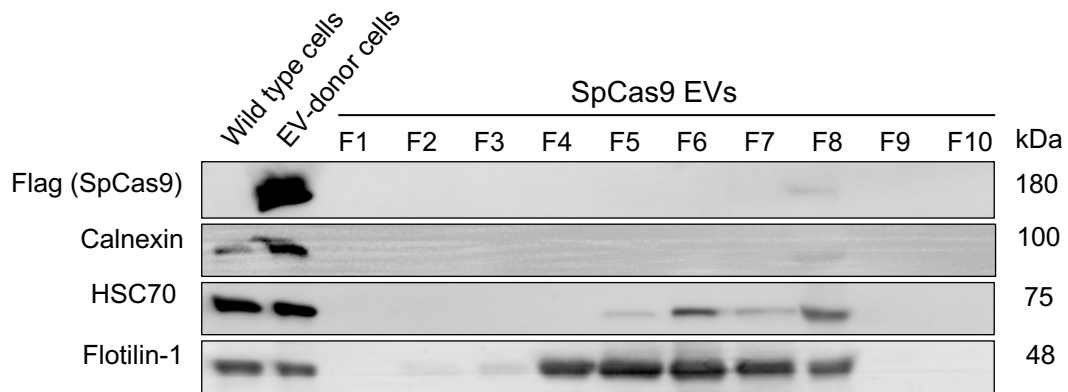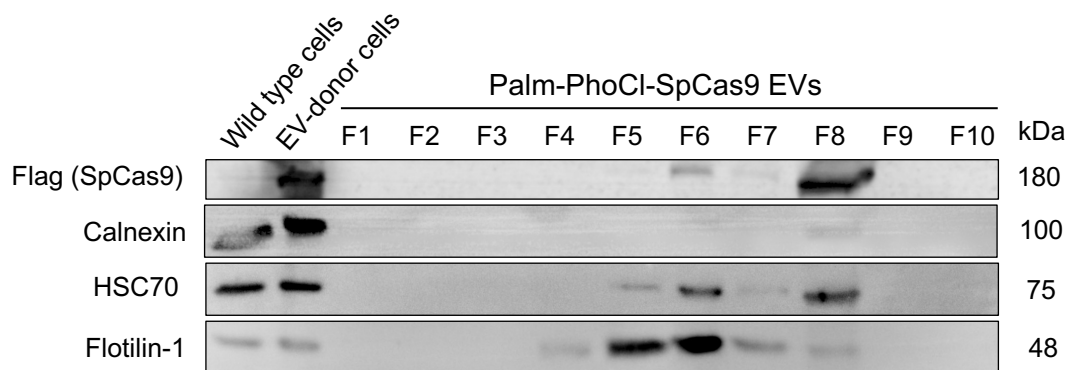

Supplement: 8 [file NIHMS2179600-supplement-8.pdf]

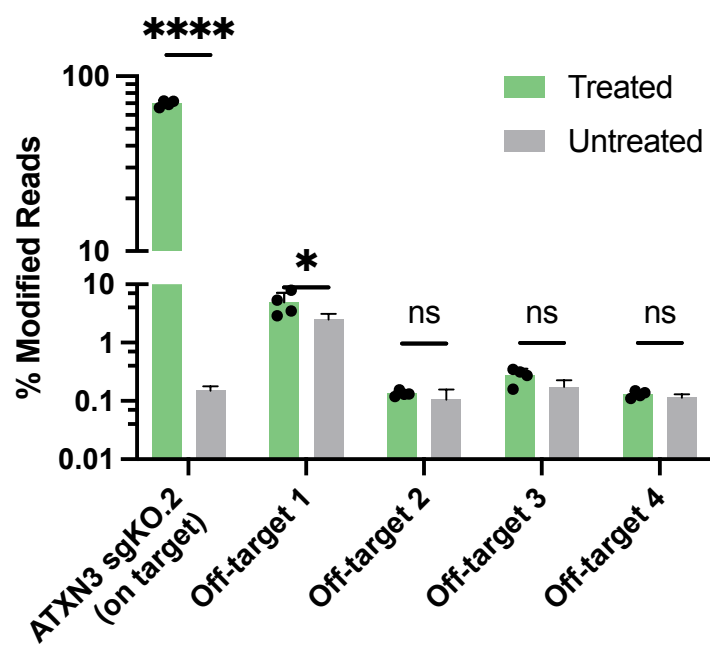

Supplement: 9 [file NIHMS2179600-supplement-9.pdf]

A.

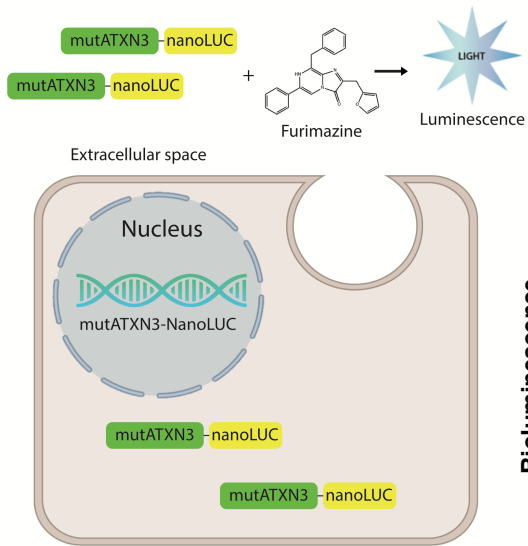

B.

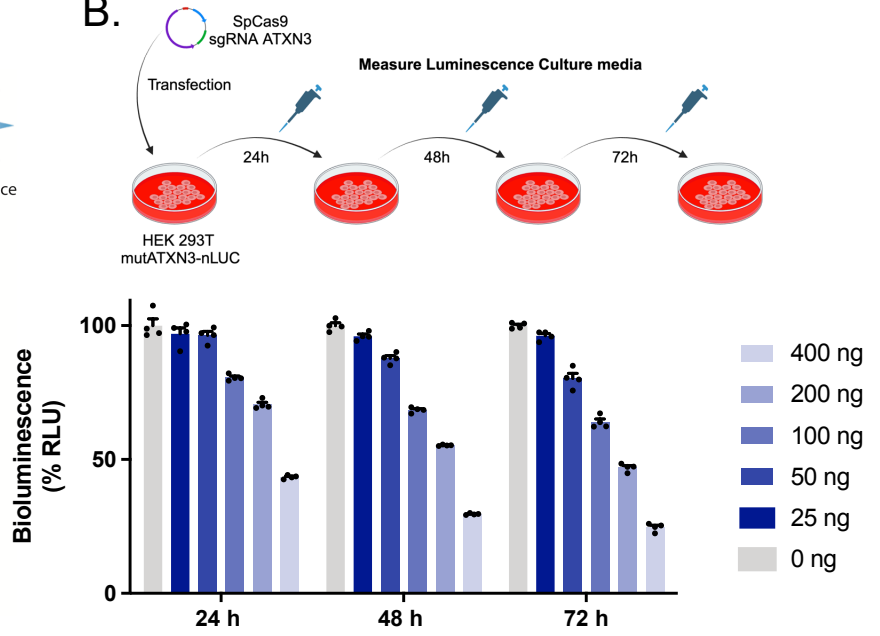

Supplement: 10 [file NIHMS2179600-supplement-10.pdf]

A.

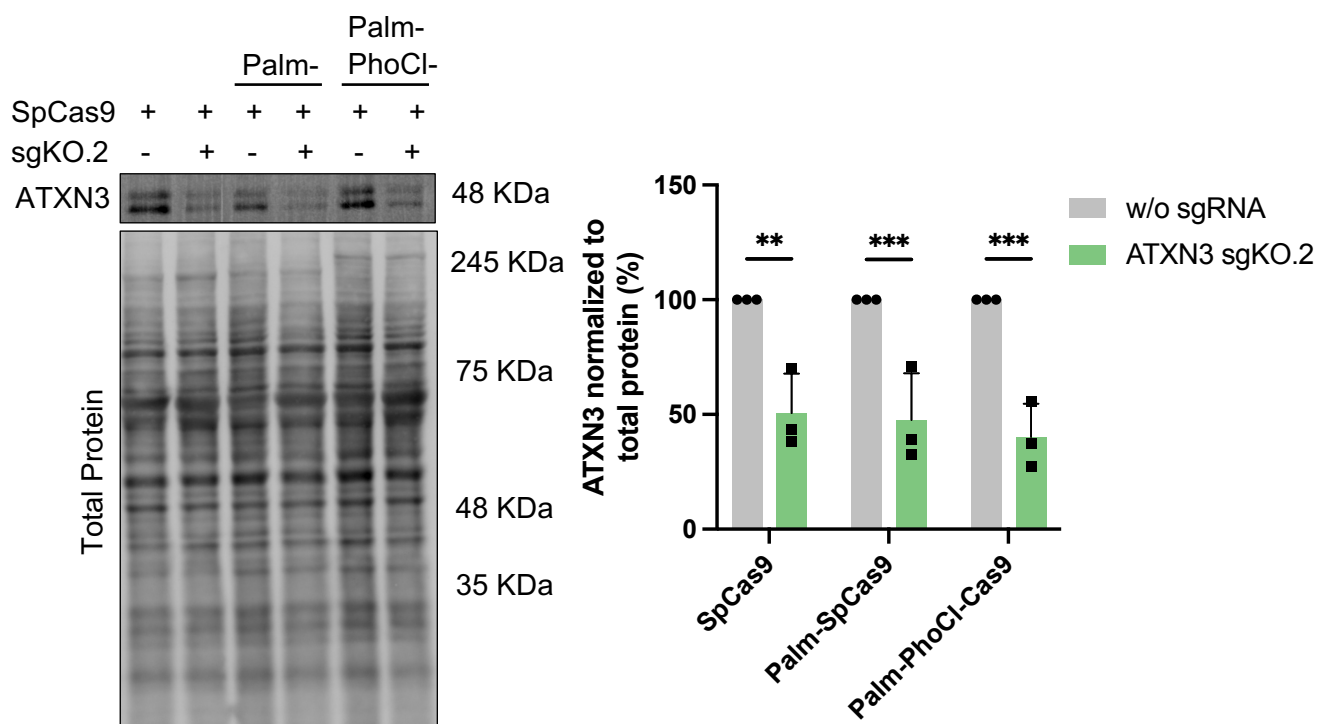

## B. Surveyor Nuclease Assay

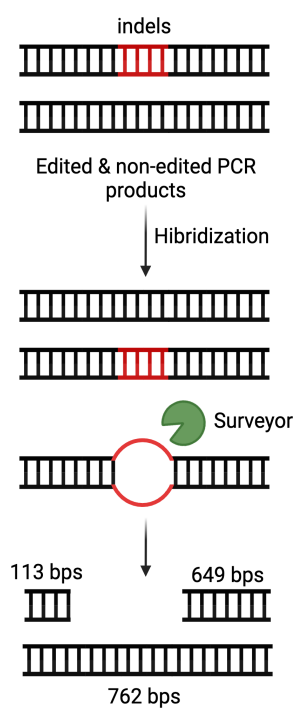

|                   |   |   |   |
|-------------------|---|---|---|
| SpCas9            | + | + | - |
| Palm-PhoCl-SpCas9 | - | - | + |
| sgKO.2            | - | + | + |

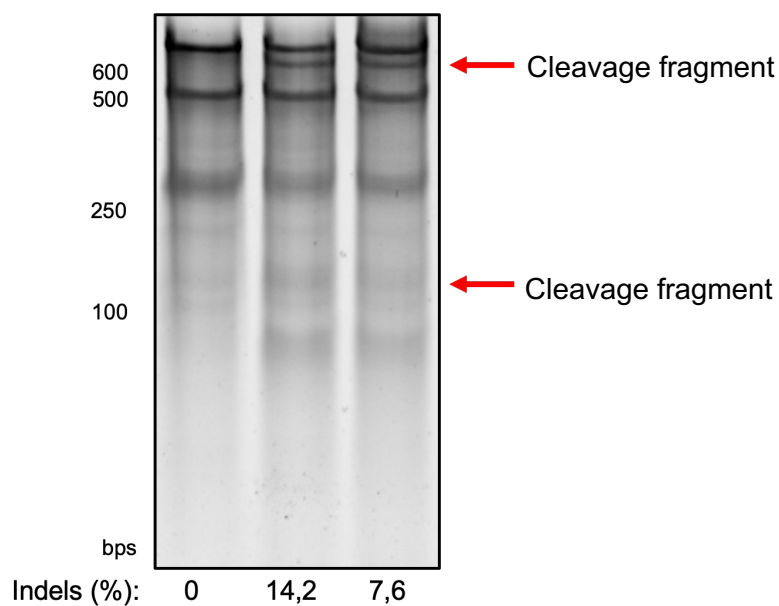

Supplement: 11 [file NIHMS2179600-supplement-11.pdf]

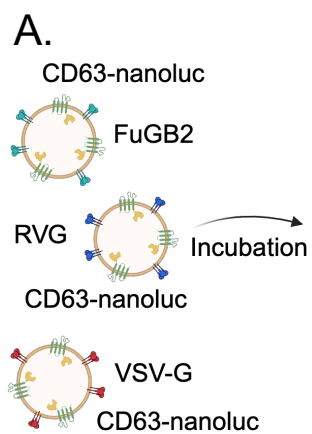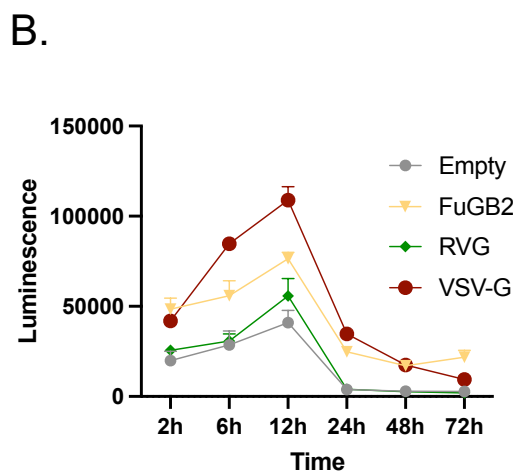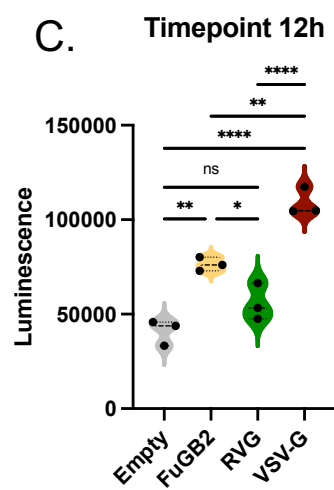

Supplement: 12 [file NIHMS2179600-supplement-12.pdf]
