# Supplementary material for: Extracellular vesicles-mediated delivery of SpCas9 RNPs for therapeutic gene editing in Spinocerebellar Ataxia Type 3
Source: Biomaterials. Author manuscript; Available in PMC 2026 Jun 23. (PMC13288182; doi:10.1016/j.biomaterials.2026.124119)
Supplement: 4 [file NIHMS2179600-supplement-4.docx]

**Supplementary Table 1: SgRNA-dependent off-target sites** **predicted using Cas-OFFinder**

| **Name** | **Target** | **Sequence** | **Chromosome** | **Position** | **Direction** | **Mismatches** |
| --- | --- | --- | --- | --- | --- | --- |
| Off-target 1 | crRNA | GTGCCTGAATAACTTATTGCANGG | chr15 | 45530579 | + | 3 |
|  | DNA | caGCCTGAATAACTTcTTGCATGG |  |  |  |  |
| Off-target 2 | crRNA | GTGCCTGAATAACTTATTGCANGG | chr22 | 40208795 | - | 3 |
|  | DNA | GTGCCTGAATtACTTtTTGgATGG |  |  |  |  |
| Off-target 3 | crRNA | GTGCCTGAATAACTTATTGCANGG | chr2 | 116457374 | + | 3 |
|  | DNA | GTtCCTGAATcACTTATTGaAAGG |  |  |  |  |
| Off-target 4 | crRNA | GTGCCTGAATAACTTATTGCANGG | chr4 | 176326889 | - | 3 |
|  | DNA | GTGCCTtcATAACTTAaTGCAAGG |  |  |  |  |

**Supplementary Table 2. Primer sequences used in PCR for NGS**

| **Name** | **Primer** | **Sequence** | **Annealing Temperature** | **Amplicon** |
| --- | --- | --- | --- | --- |
| Off-target 1 | FRW | CTCACTATGTTGCCCAGGCTG | 66 ºC | CTCACTATGTTGCCCAGGCTGGTCTTGAACTCCTGGGCTGAAGTGATCCTCCTGTCTCAGCCTCCCAAGTGCTGGGATTACAGGCACGAGCCACCAAGCCCAGCCTGAATAACTTCTTGCATGGCAGATGGAAAAGGCTGCCAACCATTTAGCTAGTCTCTCAACCAGGCAACTCGTAATATGCAGATTTAGGCAATAGCAGAAG |
|  | REV | CTTCTGCTATTGCCTAAATCTGCA |  |  |
| Off-target 2 | FRW | AGCTGAGTGGAGGGTATATAAA | 63 ºC | AGCTGAGTGGAGGGTATATAAAAGTTATTTTGTATGTATTAAATATGTAATAAAAATTTTTCTCCAGAAGTTTTATTATTTTTTAAGAGTGGAAAAATAACACAAATGTCATCTCCATCCAAAAAGTAATTCAGGCACTGAGTACTTATTTTTAAATGCGTTTTGAATTCTTTGCCAATGAGAGATGCCTGAAACACGGAAAGGAAAGACCTAGTGATCCTTGTTTGTCCCATGACATTGGC |
|  | REV | GCCAATGTCATGGGACAAAC |  |  |
| Off-target 3 | FRW | CACACTCTGAGGACTCACAG | 64 ºC | CACACTCTGAGGACTCACAGACCTTTGCCTGACTCATGGTGTAGGCTGCAGCCTGGTACTTCCTTCAAAGGGCCTGTGGATTCTTTCAGTTTCCTGTTCAGTTCCTGAATCACTTATTGAAAGGAAGTGTTTATGGCGTGAATTTCTAAACACTATTTTGTCCTTCCAAGTGGGAGAGGTGTGCTAGCAATGCCTCTAATCAGCCATGATG |
|  | REV | CATCATGGCTGATTAGAGGC |  |  |
| Off-target 4 | FRW | CTCAGAGAGAAGAATGACAATTCC | 63 ºC | CTGAGCTGGAGGGTCTTAGAAATTTTATAGGGAAAAGTGATTTAAAAGAATACTTCCCTTCTAGGGATTTAATTCATCTGTTCCTCCAAAGGCCTCACTTTTTGGAGATCTTATAGATCAAAAACCTTGCATTAAGTTATGAAGGCACTAAGTAATACAGTTGAGTTAGCAGAAGGAATGAACTCCTTCAGGGAATTGTCATTCTTCTCTCTGAG |
|  | REV | CTGAGCTGGAGGGTCTTAG |  |  |
